# Supplementary material for: Short internal open reading frames repress the translation of N-terminally truncated proteoforms
Source: EMBO Rep. 2025 Feb 17;26(6):1566–89. doi: 10.1038/s44319-025-00390-z (PMC11933307; doi:10.1038/s44319-025-00390-z)
Supplement: Supplementary file 3 — Appendix [file 44319_2025_390_MOESM3_ESM.pdf]

Appendix

Appendix Figure S1……..………………………………………………………………….2
Appendix Figure S2…………………………………………………………..……………….5

Appendix Table S1-S4………………...………………………………………………………….6

Trip6 mRNA (NM_011639.3)
UCCAAAAUUGGGGAGGAAGGGAGGAAAAAAAGCCAGAAAAAGUUUCUUUGCG
GGAGUCCCAAACGGGGCGUGGGACAGUAAGGGAGGGAAAGGGCCCAAGGCUC
CGAGCUCCAGGCUCUGAGCUUGUGGGGACCCUGACCUCUGUCUGGAGUGGAGU
AACCCUAUUCCAGACCAUGUCCGGGCCCACCUGGCUUCCCCCGAAGCAGCCAG
AACCCUCCAGACUCCCUCAGGGGAGAUCGCUGCCCAGAGGCGCCCUGGGCCCG
CCAACGGCCCACGGAGCAAcacuccagccucaccccagggucaacuuuugccccucccgccugaacacug
uuaucagccuccgggggguaccggaagaucgggggccuacuugggugggaucccauggaacaccccagcgccugcagGG
GCUCCCUCCAGACAGGGGGAUCAUCCGCCCUGGCAGUCUGGAUGCUGAGAUAG
AUUCGCUCACCAGCAUGUUGGCUGAUCUGGACGGGGGUCGCAGUCAUGCACCU
AGGCGGCCAGACAGACAGgcuuuugaggcucccccaccccaugcuuaccgcggaggcucccugaagcccag
uggaggugcuguuccaaccccgaugcucccagcaucccacuaugguggaccuaccccagccuccuaugcuaccgcgagca
cgccagcuggcccugcuuucccuguacaagugaaggugggcucaaccugugagaggcuguggacugcccaggcgagggggc
cucucaggccucugggccucuuccaggcccccacuuuccucugacaggucguggugaagucugggggggcuggcuauagg
agccaccgagagccaggaccgggggguuccggagggaccuucuggaguacauaucccugcaggaggagggagaggaggug
ggcaugagccucagGGCCCCUUAGGCCAACCUCCUGAAGAGGAACUGGAGAGACUGA
CCAAGAAACUGGUGCAUGACAUGAGCCACCCUCCCAGUGGGGAGUACUUUGguc
ggugugguggcuguggcgaagauguggugggcgauggagcuggggguuguggcccuggaccgugucuuccauauuggu
ugcuuuguguguucuaccugucgggcccagcuccggggccagcacuucuaugcuguggagaggcgggcauauugugag
agcugcuaugugGCCACCCUGGAGAAAUGUUCCACAUGCUCUGAACCCAUCCUGGAC
CGAAUCCUGAGGGCUAUGGGGAAGGCGUACCACCCUGGUUGCUUCACCUGUGU
GGUAUGCCACCGUGGUCUUGAUGGCAUCCCGUUCACAGUGGACGCCACCAGCC
AGAUCCACUGCAUUGAAGAUUUCCACAGgaaauuugccccacgaugcucagugugugguggggcc
aucaugccggaaccaggucaggaggagacggugagaauuguugcucuggaucgaaguuuccacaucggcuguuacaagu
gugagGAGUGUGGGCUGCUGCUGUCCUCUGAGGGAGAGUGUCAAGGCUGCUACC
CGCUGGAUGGGCACAUCUUGUGCAAGGCUUGCAGCGCCUGGCGUAUCCAAGAG
CUCUCAGCCACUGUCACCACUGAUUGU**UGA**CUGCUCUGAGAAGUACCUGCUGG
GAUCUCAGCCCCCGUCCGUCCGUCCGUCACUUCCCCCAACAUCUACCCUUAGU
GUUUGCCAUCAAAUGCUGUCUUUUCUUCCUCUCUUCAUGAAAUAAUAAUCCCU
GGAGCAUUUAAAAGC

Splice variant 1:
uccaaaauuggggaggaagggaggaaaaaaagccagaaaaaguuucuuugcgggagucccaaacggggcgugggacagu
aagggagggaaagggcccaaggcuccgagcuccaggcucugagcuuguggggacccugaccucugucuggaguggagua
acccuauuccagaccaugucccgggcccaccuggcuucccccgaagcagccagaacccuccagacucccucaggggagauc
gcugcccagaggcgcccugggcccgccaacggcccacggagcaaCACUCCAGCCUCACCCCAGGGUCA
ACUUUUGCCCCCUCCCGCCUGAACACUGUUAUCAGCCUCCGGGGGUACCGGAA
GAUCGGGGGCCUACUUGGGUGGGAUCCCAUGGAACACCCCAGCGCCUGCAGggg
cucccuccagacagggggaucauccgcccuggcagucuggaugcugagauagauucgcucaccagcauguuggcugauc
uggacgggggucgcagucaugcaccuaggcggccagacagacagGCCACCCUGGAGAAAUGUUCCAC
AUGCUCUGAACCCAUCCUGGACCGAAUCCUGAGGGCUAUGGGGAAGGCGUACC
ACCCUGGUUGCUUCACCUGUGUGGUAUGCCACCGUGGUCUUGAUGGCAUCCCG
UUCACAGUGGACGCCACCAGCCAGAUCCACUGCAUUGAAGAUUUCCACAGgaaau
uugccccacgaugcucagugugugguggggccaucaugccggaaccaggucaggaggagacggugagaauuguugcuc
uggaucgaaguuuccacaucggcuguuacaagugugagGAGUGUGGGCUGCUGCUGUCCUCUGAG
GGAGAGUGUCAAGGCUGCUACCCGCUGGAUGGGCACAUCUUGUGCAAGGCUU
GCAGCGCCUGGCGUAUCCAAGAGCUCUCAGCCACUGUCACCACUGAUUGU**UGA**
CUGCUCUGAGAAGUACCUGCUGGGAUCUCAGCCCCCGUCCGUCCGUCCGUCAC
UUCCCCCAACAUCUACCCUUAGUGUUUGCCAUCAAAUGCUGUCUUUUCUUCCU
CUCUUCAUGAAAUAAUAAUCCCUGGAGCAUUUAAAAGC

Splice variant 2:
uccaaaauuggggaggaagggaggaaaaaaagccagaaaaguuucuuugcgggagucccaaacggggcgugggacagu
aagggagggaaagggcccaaggcuccgagcuccaggcucugagcuuguggggacccugaccucugucuggaguggagua
acccuauuccagaccauguccgggcccaccuggcuucccccgaagcagccagaacccuccagacucccucaggggagauc
gcugcccagaggcgcccugggcccgccaacggcccacggagcaaCACUCCAGCCUCACCCCAGGGUCA
ACUUUUGCCCCCUCCCGCCUGAACACUGUUAUCAGCCUCCGGGGGUACCGGAA
GAUCGGGGGCCUACUUGGGUGGGAUCCCAUGGAACACCCCAGCGCCUGCAGggc
cccuuaggccaaccuccugaagaggaacuggagagacugaccaagaaacuggugcaugacaugagccacccucccagugg
ggaguacuuugGUCGGUGUGGUGGCUGUGGCGAAGAUGUGGUGGGCGAUGGAGCUG
GGGUUGUGGCCCUGGACCGUGUCUUCCAUAUUGGUUGCUUUGUGUGUUCUAC
CUGUCGGGCCCAGCUCCGGGGCCAGCACUUCUAUGCUGUGGAGAGGCGGGCAU
AUUGUGAGAGCUGCUAUGUGgccacccuggagaaauguuccacaugcucugaacccauccuggaccgaa
uccugagggcuaugggaaggcguaccacccugguugcuucaccugugugguaugccaccguggucuugauggcaucc
cguucacaguggacgccaccagccagauccacugcauugaagauuuccacagGAAAUUUGCCCCACGAUG
CUCAGUGUGUGGUGGGGCCAUCAUGCCGGAACCAGGUCAGGAGGAGACGGUG
AGAAUUGUUGCUCUGGAUCGAAGUUUCCACAUCGGCUGUUACAAGUGUGAGga
gugugggcugcugcuguccucugagggagagugucaaggcugcuacccgcuggaugggcacaucuugugcaaggcuug
cagcgccuggcguauccaagagcucucagccacugucaccacugauugu**UGA**cugcucugagaaguaccugcugggau
cucagcccccguccguccguccgucacuucccccaacaucuacccuuaguguuugccaucaaaugcugucuuuucuuccu
cucuucaugaaauaauaaucccuggagcauuuaaaagc

Splice variant 3:
uccaaaauuggggaggaagggaggaaaaaaagccagaaaaguuucuuugcgggagucccaaacggggcgugggacagu
aagggagggaaagggcccaaggcuccgagcuccaggcucugagcuuguggggacccugaccucugucuggaguggagua
acccuauuccagaccauguccgggcccaccuggcuucccccgaagcagccagaacccuccagacucccucaggggagauc
gcugcccagaggcgcccugggcccgccaacggcccacggagcaaCACUCCAGCCUCACCCCAGGGUCA
ACUUUUGCCCCCUCCCGCCUGAACACUGUUAUCAGCCUCCGGGGGUACCGGAA
GAUCGGGGGCCUACUUGGGUGGGAUCCCAUGGAACACCCCAGCGCCUGCAGggg
cucccuccagacagggggaucauccgcccuggcagucuggaugcugagauagauucgcucaccagcauguuggcugauc
uggacgggggucgcagucaugcaccuaggcggccagacagacagGUCGUGGUGAAGUCUGGGGGGCU
GGCUAUAGGAGCCACCGAGAGCCAGGACCGGGGGUUCCGGAGGGACCUUCUGG
AGUACAUAUCCCUGCAGGAGGAGGGAGAGGAGGUGGGCAUGAGCCUCAGggccc
cuuaggccaaccuccugaagaggaacuggagagacugaccaagaaacuggugcaugacaugagccacccucccaguggggg
aguacuuugGUCGGUGUGGUGGCUGUGGCGAAGAUGUGGUGGGCGAUGGAGCUGG
GGUUGUGGCCCUGGACCGUGUCUUCCAUAUUGGUUGCUUUGUGUGUUCUACC
UGUCGGGCCCAGCUCCGGGGCCAGCACUUCUAUGCUGUGGAGAGGCGGGCAUA
UUGUGAGAGCUGCUAUGUggccacccuggagaaauguuccacaugcucugaacccauccuggaccgaaucc
ugagggcuaugggaaggcguaccacccugguugcuucaccugugugguaugccaccguggucuugauggcaucccgu
ucacaguggacgccaccagccagauccacugcauugaagauuuccacagGAAAUUUGCCCCACGAUGCUC
AGUGUGUGGUGGGGCCAUCAUGCCGGAACCAGGUCAGGAGGAGACGGUGAGA
AUUGUUGCUCUGGAUCGAAGUUUCCACAUCGGCUGUUACAAGUGUGAGgagugu
gggcugcugcuguccucugagggagagugucaaggcugcuacccgcuggaugggcacaucuugugcaaggcuugcagc
gccuggcguauccaagagcucucagccacugucaccacugauugu**uga**cugcucugagaaguaccugcugggaucucag
cccccguccguccguccgucacuucccccaacaucuacccuuaguguuugccaucaaaugcugucuuuucuuccucucuu
caugaaauaauaaucccuggagcauuuaaaagc

5’ RACE clone 1
UCCAAAAUUGGGGAGGAAGGGAGGAAAAAAAGCCAGAAAAAGUUUCUUUGCG
GGAGUCCCAAACGGGGCGUGGGACAGUAAGGGAGGGAAAGGGCCCAAGGCUC
CGAGCUCCAGGCUCUGAGCUUGUGGGGACCCUGACCUCUGUCUGGAGUGGAGU
AACCCUAUUCCAGACCAUGUCCGGGCCCACCUGGCUUCCCCCGAAGCAGCCAG
AACCCUCCAGACUCCCUCAGGGGAGAUCGCUGCCCAGAGGCGCCCUGGGCCCG
CCAACGGCCCACGGAGCAAgcccccacuuuccucugacaggucguggugaagucugggggcuggcuaua
ggagccaccgagagccaggaccgggggguuccggagggaccuucuggaguacauaucccugcaggaggagggagaggagg
ugggcaugagccucagGGCCCCUUAGGCCAACCUCCUGAAGAGGAACUGGAGAGACUG
ACCAAGAAACUGGUGCAUGACAUGAGCCACCCUCCCAGUGGGGAGUACUUUGg
ucggugugguggcuguggcgaagaugguggggcgauggagcuggggguuguggcccuggaccgugucuuccauauug
guugcuuuguguguucuaccugucgggcccagcuccggggccagcacuucuaugcuguggagaggcgggcauauugug
agagcugcuaugugGCCACCCUGGAGAAAUGUUCCACAUGCUCUGAACCCAUCCUGG
ACCGAAUCCUGAGGGCUAUGGGGAAGGCGUACCACCCUGGUUGCUUCACCUGU
GUGGUAUGCCACCGUGGUCUUGAUGGCAUCCCGUUCACAGUGGACGCCACCAG
CCAGAUCCACUGCAUUGAAGAUUUCCACAGgaaauuugccccacgaugcucagugugugguggg
gccaucaugccggaaccaggucaggaggagacggugagaauuguugcucuggaucgaaguuuccacaucggcuguuaca
agugugagGAGUGUGGCUGCUGCUGUCCUCUGAGGGAGAGUGUCAAGGCUGCUA
CCCGCUGGAUGGGCACAUCUUGUGCAAGGCUUGCAGCGCCUGGCGUAUCCAAG
AGCUCUCAGCCACUGUCACCACUGAUUGUUGACUGCUCUGAGAAGUACCUGCU
GGGAUCUCAGCCCCCGUCCGUCCGUCCGUCACUUCCCCCAACAUCUACCCUUA
GUGUUUGCCAUCAAAUGCUGUCUUUUCUUCCUCUCUUCAUGAAAUAAUAAUCC
CUGGAGCAUUUAAAAGC

5’ RACE clone 2
ucccuguacaagugaaggugg cucaaccugugagaggcuguggacugcccaggcgagggg ccucucaggccucugggcc
ucuuccaggcccccacuuuccucugacaggucguggugaagucugggggcuggcuauaggagccaccgagagccagga
ccgggggguuccggagggaccuucuggaguacauaucccugcaggaggagggagaggaggugggcaugagccucagGG
CCCCUUAGGCCAACCUCCUGAAGAGGAACUGGAGAGACUGACCAAGAAACUGG
UGCAUGACAUGAGCCACCCUCCCAGUGGGGAGUACUUUGgucggugugguggcuguggc
gaagaugguggggcgauggagcuggggguuguggcccuggaccgugucuuccauauugguugcuuuguguguucuac
cugucgggcccagcuccggggccagcacuucuaugcuguggagaggcgggcauauugugagagcugcuaugugGCC
ACCCUGGAGAAAUGUUCCACAUGCUCUGAACCCAUCCUGGACCGAAUCCUGAG
GGCUAUGGGGAAGGCGUACCACCCUGGUUGCUUCACCUGUGUGGUAUGCCACC
GUGGUCUUGAUGGCAUCCCGUUCACAGUGGACGCCACCAGCCAGAUCCACUGC
AUUGAAGAUUUCCACAGgaaauuugccccacgaugcucagugugugguggggccaucaugccggaaccag
gucaggaggagacggugagaauuguugcucuggaucgaaguuuccacaucggcuguuacaagugugagGAGUGUG
GGCUGCUGCUGUCCUCUGAGGGAGAGUGUCAAGGCUGCUACCCGCUGGAUGGG
CACAUCUUGUGCAAGGCUUGCAGCGCCUGGCGUAUCCAAGAGCUCUCAGCCAC
UGUCACCACUGAUUGUUGACUGCUCUGAGAAGUACCUGCUGGGAUCUCAGCCC
CCGUCCGUCCGUCCGUCACUUCCCCCAACAUCUACCCUUAGUGUUUGCCAUCA
AAUGCUGUCUUUUCUUCCUCUCUUCAUGAAAUAAUAAUCCCUGGAGCAUUUA
AAAGC

Appendix Figure S1. Annotated sequences of *Trip6* mRNA and of the identified *Trip6* mRNA variants.
Exons are depicted in alternating case. The main features are color-coded as follows: Initiation codon; In frame stop codon; Nuclear Export Signal; Interaction domains; LIM domain 1, LIM domain 2; LIM domain 3

A

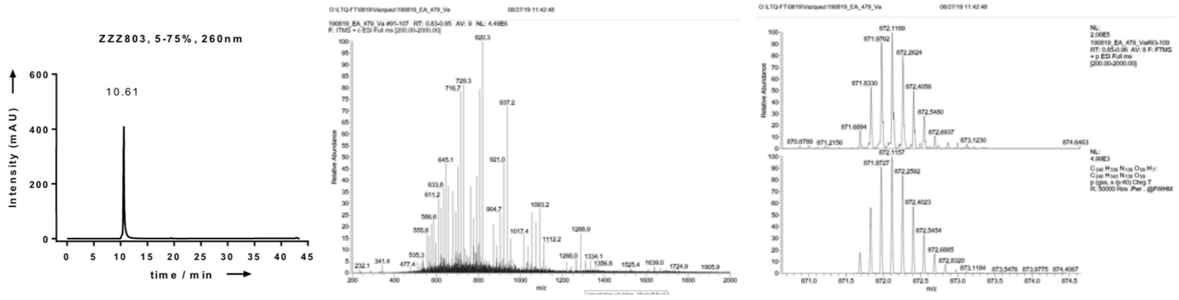

$C_{240}H_{336}N_{138}O_{59}$ [6098.19]

m/z = 555.8 (M+11H), 586.6 (M+8H+3TFA), 611.2 (M+10H), 633.8 (M+8H+2TFA), 645.1 (M+7H+3TFA), 716.7 (M+6H+3TFA), 729.3 (M+5H+4TFA), 820.3 (M+4H+4TFA), 904.7 (M+5H+2TFA), 921.0 (M+4H+3TFA), 937.2 (M+3H+4TFA), 1017.4 (M+6H), 1093.2 (M+2H+4TFA), 1112.2 (M+1H+5TFA), 1266.0 (M+3H+2TFA), 1288.9 (M+2H+3TFA), 1334 (M+5TFA)

B

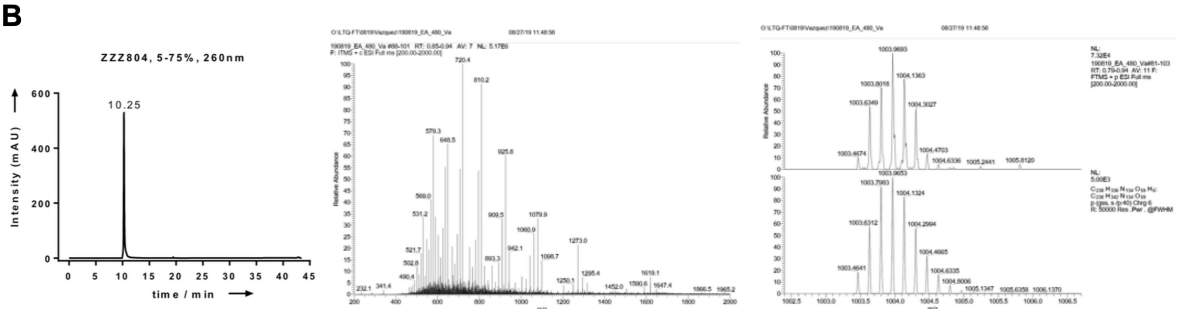

$C_{238}H_{336}N_{134}O_{59}$ [6018.14]

m/z = 490.4 (M+10H+3TFA), 502.8 (M+12H), 521.7 (M+10H+2TFA), 531.2 (M+9H+3TFA), 569.0 (M+9H+2TFA), 579.3 (M+8H+3TFA), 648.5 (M+6H+4TFA), 720.4 (M+5H+4TFA), 810.2 (M+4H+4TFA), 893.3 (M+5H+2TFA), 909.5 (M+4H+3TFA), 925.8 (M+3H+4TFA), 942.1 (M+2H+5TFA), 1060.9 (M+3H+3TFA), 1079.9 (M+2H+4TFA), 1098.7 (M+1H+5TFA), 1250.1 (M+3H+2TFA), 1273.0 (M+2H+3TFA), 1295.4 (M+1H+4TFA), 1619.1 (M+4TFA)

Appendix Figure S2. HPLC and high-resolution mass spectrometry analysis of the cell-penetrating AUG2 targeting (A) and mismatched control (B) peptide nucleic acids.

Appendix Table S1: Cell-penetrating peptide nucleic acids (PNAs)

	Sequence 5' to 3'
Trip6 AUG2	NH_2-GTCCAGATCAGCCAACAT-R_8-$CONH_2$
Misp*	NH_2-CTCCACATCACCCAAGAT-R_8-$CONH_2$
Random	NH_2-AACTGACTGAAAGGCCTC-R_8-$CONH_2$

The cell-penetrating moiety of the PNAs consists of an octoarginine peptide (R8). *The mispaired bases are underlined

Appendix Table S2: Primers used for the dentification of alternative *Trip6* mRNAs

	Sequence 5' to 3'
RT-PCR primers to identify splice variants	
5' UTR forward primer	GTGGGGACCCTGACCTCTGT
Exon 9 reverse primer	TCAACAATCAGTGGTGACAG
Trip6 specific reverse primers used in RLM-RACE	
Outer primer	ACAGGTAGAACACACAAAGCAACC
Inner primer	CCAGTTTCTTGGTCAGTCTCTCCA

Table S3: Real-time PCR primers

Gene name	Forward primer (5' to 3')	Reverse primer (5' to 3')
Myog	GAGACATCCCCCTATTTCTACCA	GCTCAGTCCGCTCATAGCC
Rplp0	GGACCCGAGAAGACCTCCTT	GCACATCACTCAGAATTTCAATGG
Tnni2	CATGGAGGTGAAGGTGCAGA	CTCTTGAACTTGCCCCTCAGG
Trip6	TCGAAGTTTCCACATCGGCT	GCTCTTGGATACGCCAGGC

Table S4: Oligonucleotides used in the translation initiation assay

		Sequence 5' to 3'	
Oligo dT adapter		GCGAGCACAGAATTAATACGACTCACTATAGG(T)$_{12}$VN	
Reverse primer		GCGAGCACAGAATTAATACGAC	
Forward primers *			Position §
Trip6	AUG1	CTATTCCAGACC**ATG**TCCG	163-181
	iuAUG	GGGTGGGATCCC**ATG**GAACA	377-396
	AUG2	TCGCTCACCAGC**ATG**TTGGC	469-488
	non-init.	CGGGCATATTGTGAGAGC	1159-1176
GR	AUG	TTGCCA**ATG**GACTCCAAAGAATCC	348-371
	non-init.	AAATGGGCAAAGGCGATACCAG	2124-2145

* The initiation codons are underlined. § Positions relative to the mRNA sequences of *Trip6* (NM_011639.3) and Glucocorticoid receptor (GR; NM_008173.4).
