# Supplementary figures and images for: Short internal open reading frames repress the translation of N-terminally truncated proteoforms
Source: EMBO Rep. 2025 Feb 17;26(6):1566–89. doi: 10.1038/s44319-025-00390-z (PMC11933307; doi:10.1038/s44319-025-00390-z)

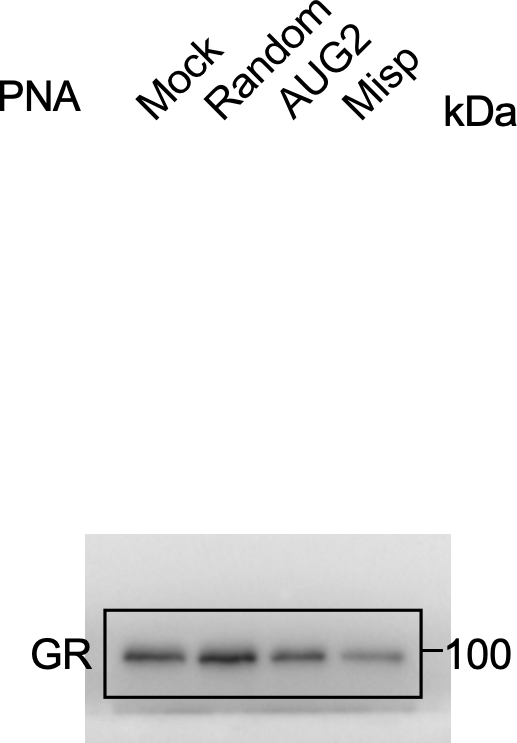

Supplement: Supplementary file 5 — Source data Fig. 1 [file 44319_2025_390_MOESM5_ESM.zip › Figure 1/1F/western_GR.tif]

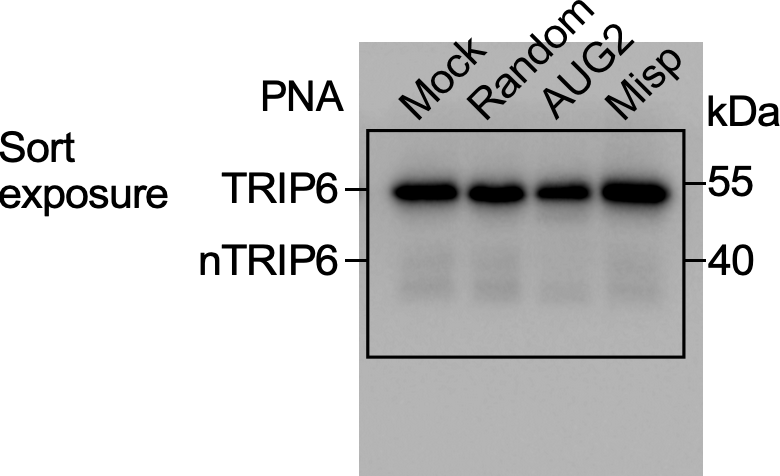

Supplement: Supplementary file 5 — Source data Fig. 1 [file 44319_2025_390_MOESM5_ESM.zip › Figure 1/1F/western_TRIP6_short_exposure.tif]

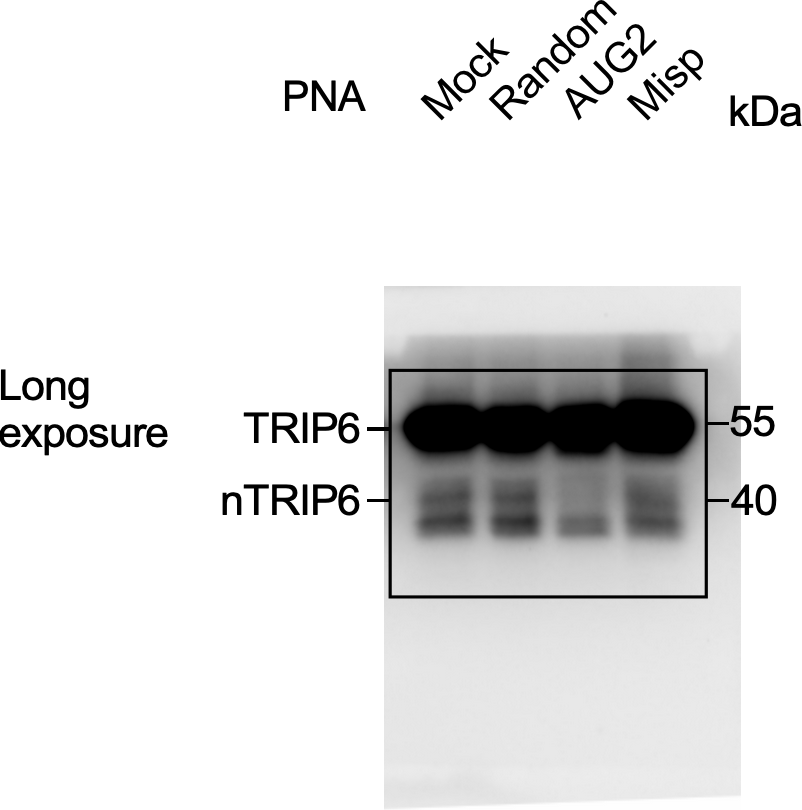

Supplement: Supplementary file 5 — Source data Fig. 1 [file 44319_2025_390_MOESM5_ESM.zip › Figure 1/1F/western_TRIP6_long_exposure.tif]

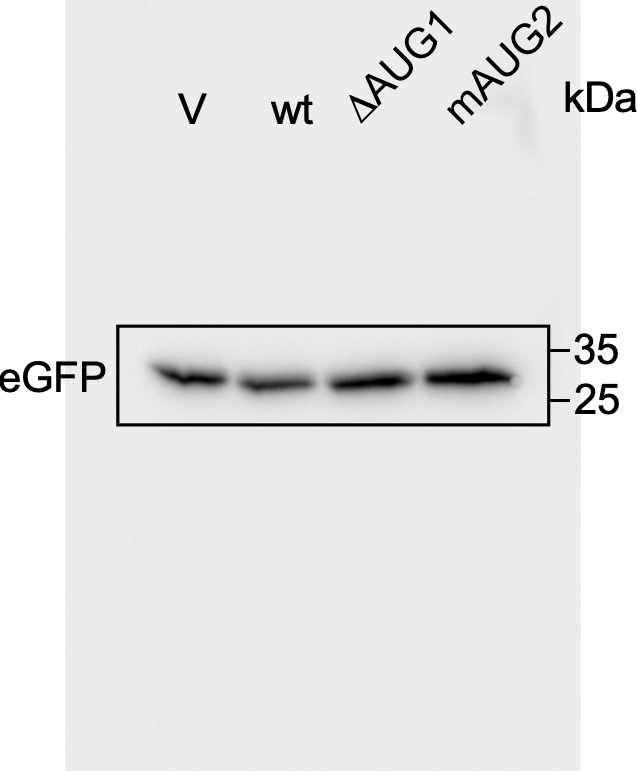

Supplement: Supplementary file 5 — Source data Fig. 1 [file 44319_2025_390_MOESM5_ESM.zip › Figure 1/1B/western_eGFP.tif]

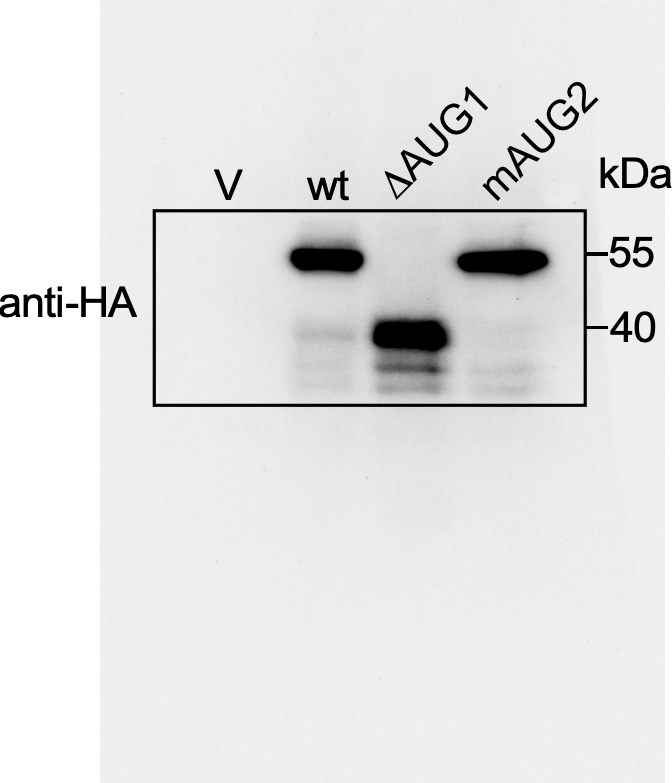

Supplement: Supplementary file 5 — Source data Fig. 1 [file 44319_2025_390_MOESM5_ESM.zip › Figure 1/1B/western_HA.tif]

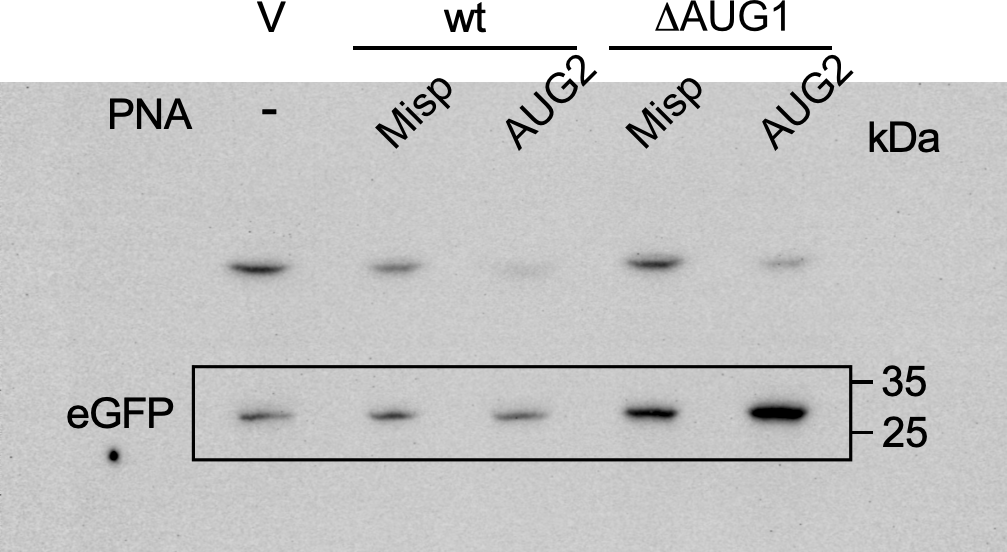

Supplement: Supplementary file 5 — Source data Fig. 1 [file 44319_2025_390_MOESM5_ESM.zip › Figure 1/1D/western eGFP.tif]

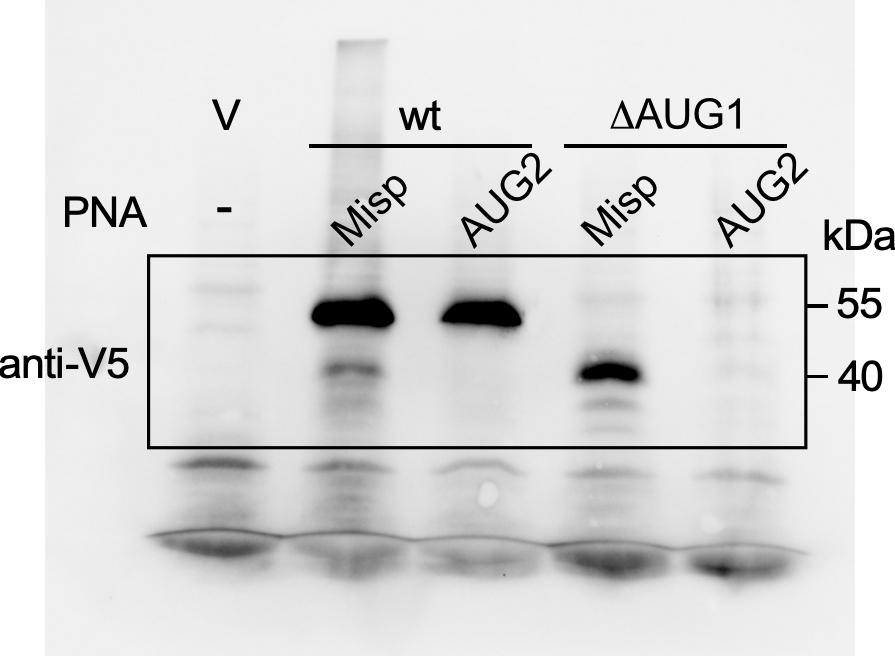

Supplement: Supplementary file 5 — Source data Fig. 1 [file 44319_2025_390_MOESM5_ESM.zip › Figure 1/1D/western V5.tif]

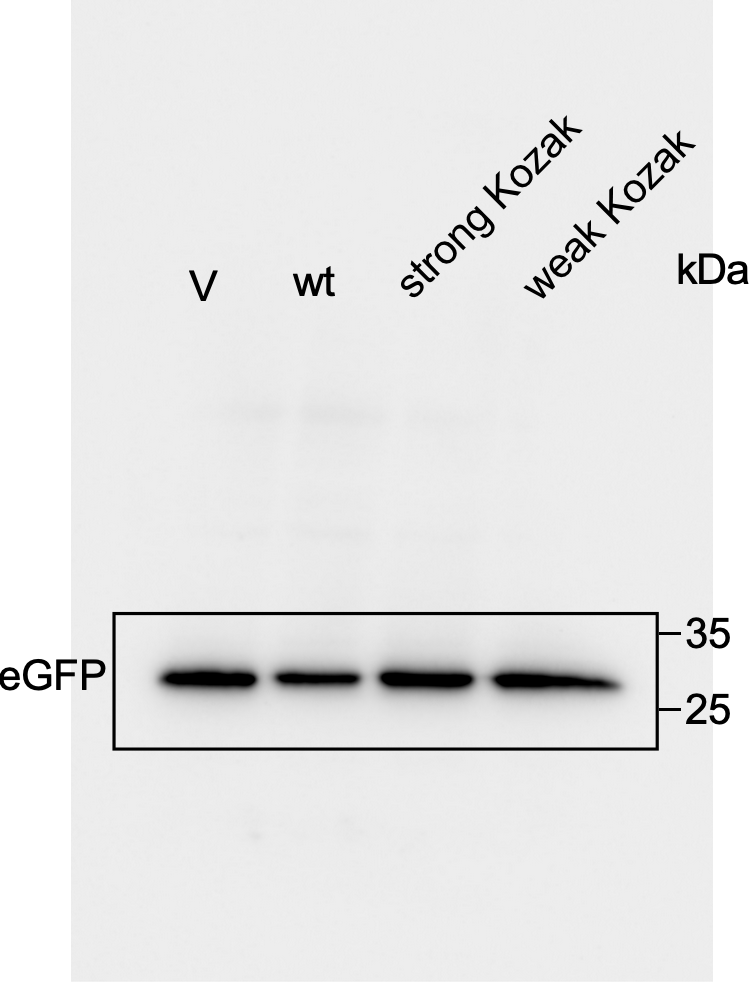

Supplement: Supplementary file 6 — Source data Fig. 2 [file 44319_2025_390_MOESM6_ESM.zip › Figure 2/2F/western_eGFP.tif]

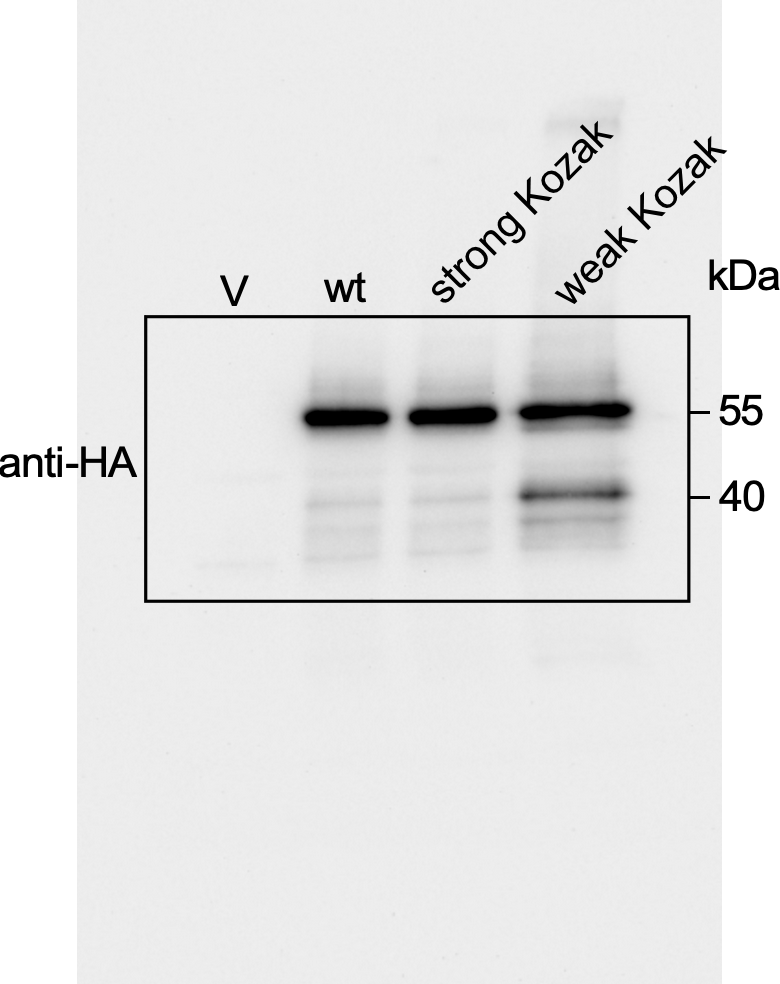

Supplement: Supplementary file 6 — Source data Fig. 2 [file 44319_2025_390_MOESM6_ESM.zip › Figure 2/2F/western_HA.tif]

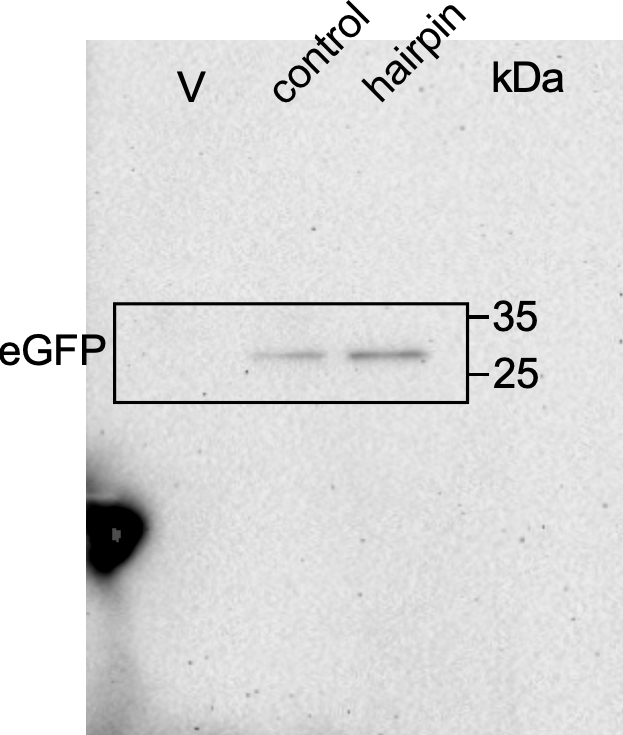

Supplement: Supplementary file 6 — Source data Fig. 2 [file 44319_2025_390_MOESM6_ESM.zip › Figure 2/2B/western eGFP.tif]

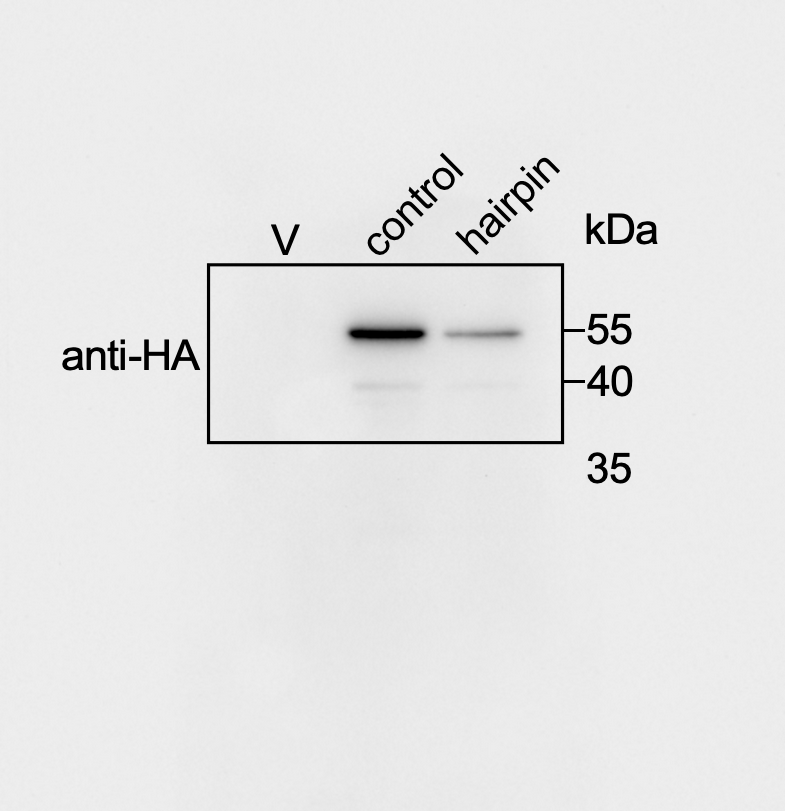

Supplement: Supplementary file 6 — Source data Fig. 2 [file 44319_2025_390_MOESM6_ESM.zip › Figure 2/2B/western HA.tif]

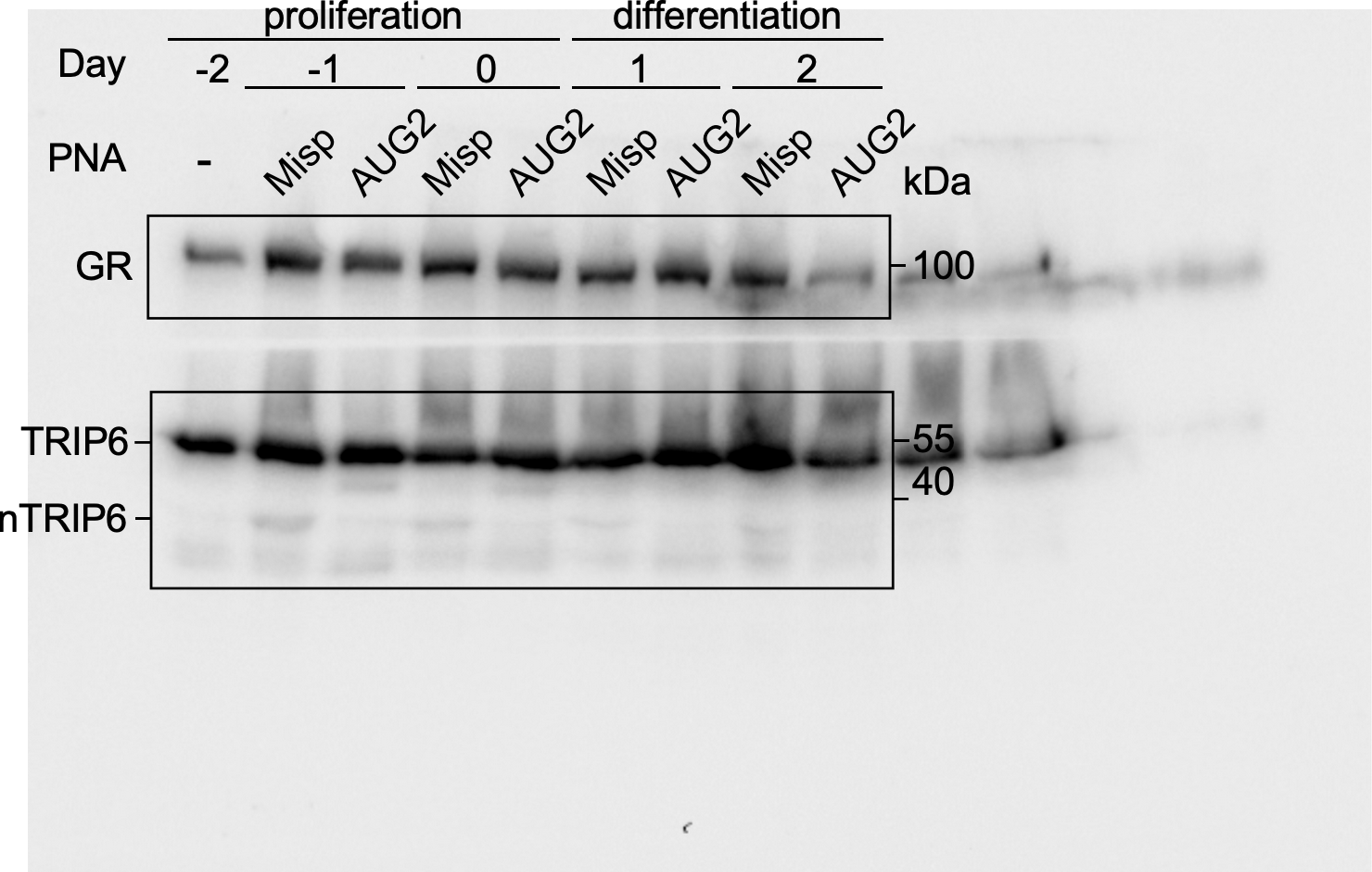

Supplement: Supplementary file 7 — Source data Fig. 3 [file 44319_2025_390_MOESM7_ESM.zip › Figure 3/3A/western_TRIP6_GR.tif]

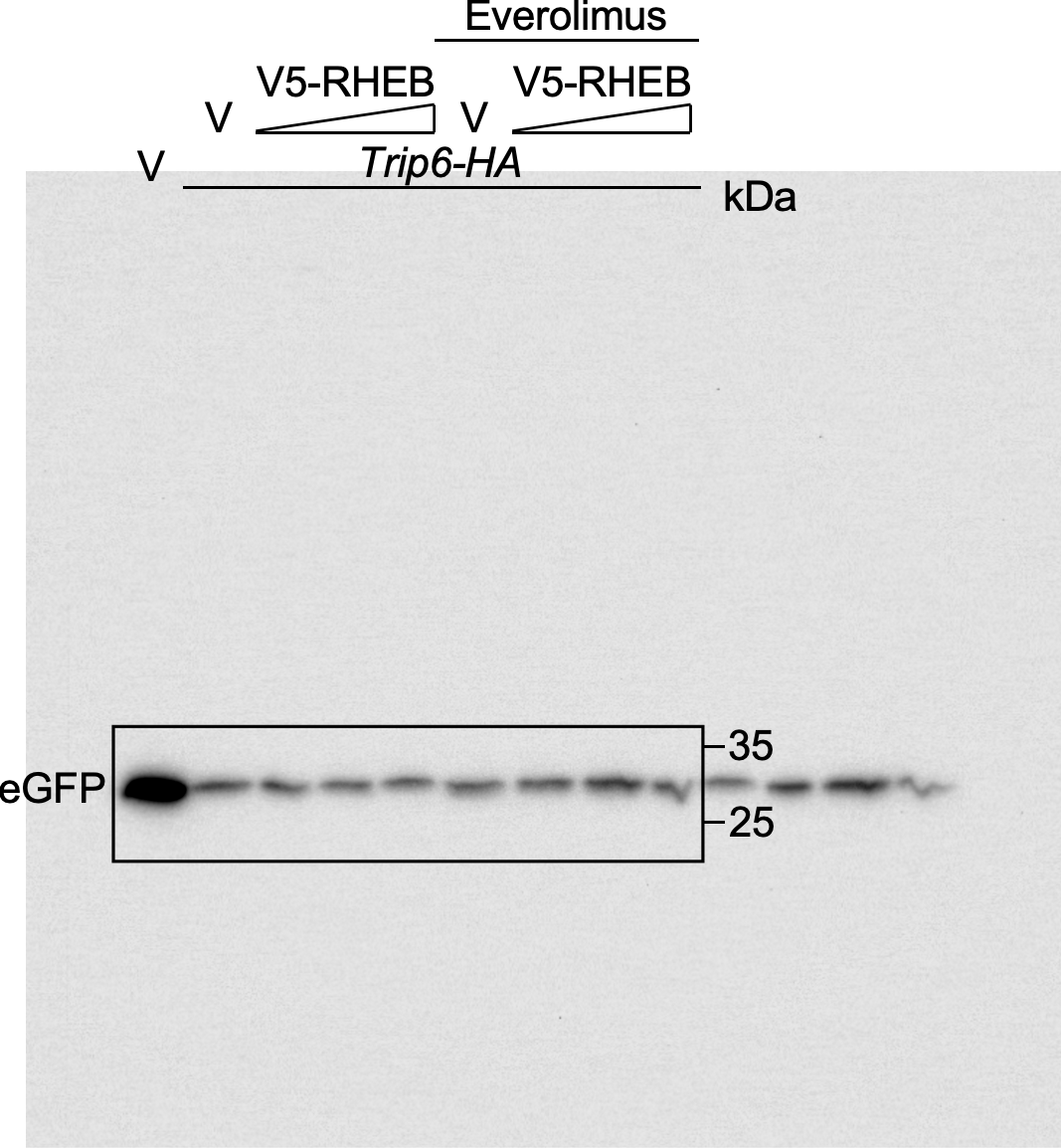

Supplement: Supplementary file 8 — Source data Fig. 4 [file 44319_2025_390_MOESM8_ESM.zip › Figure 4/4D/western_eGFP.tif]

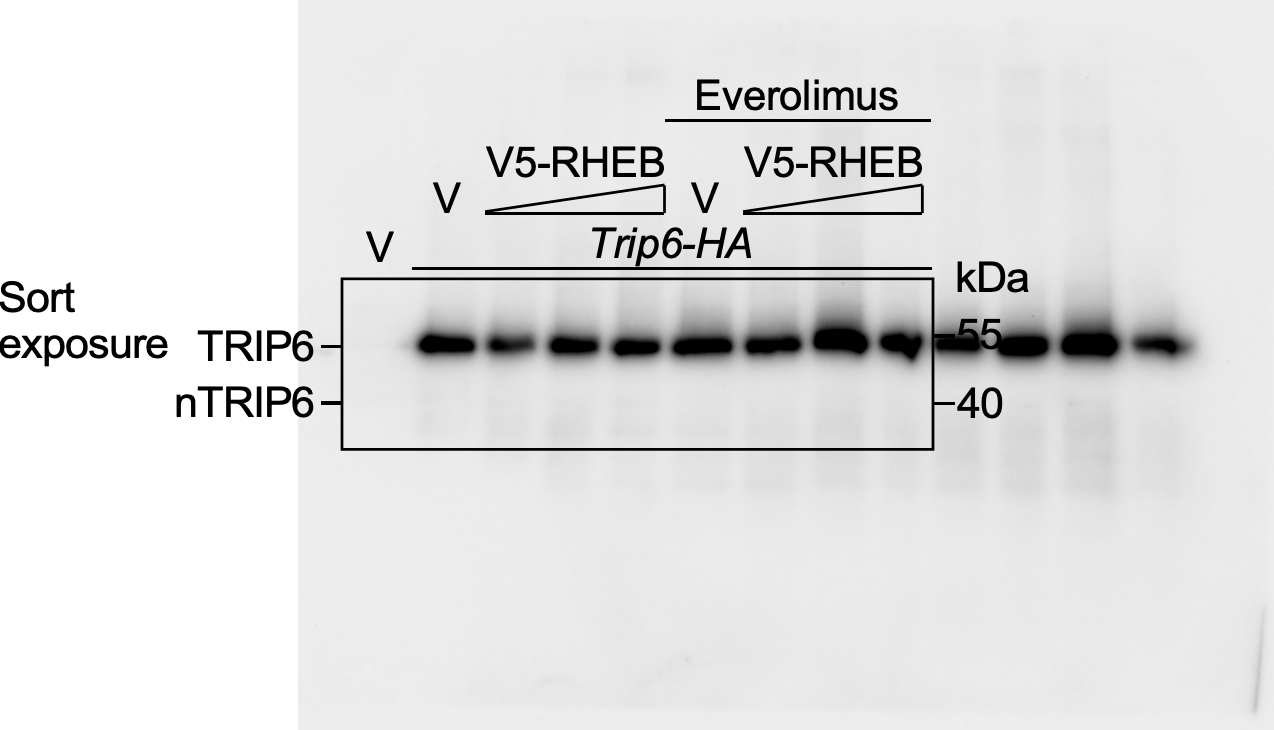

Supplement: Supplementary file 8 — Source data Fig. 4 [file 44319_2025_390_MOESM8_ESM.zip › Figure 4/4D/western_TRIP6_short_exposure.tif]

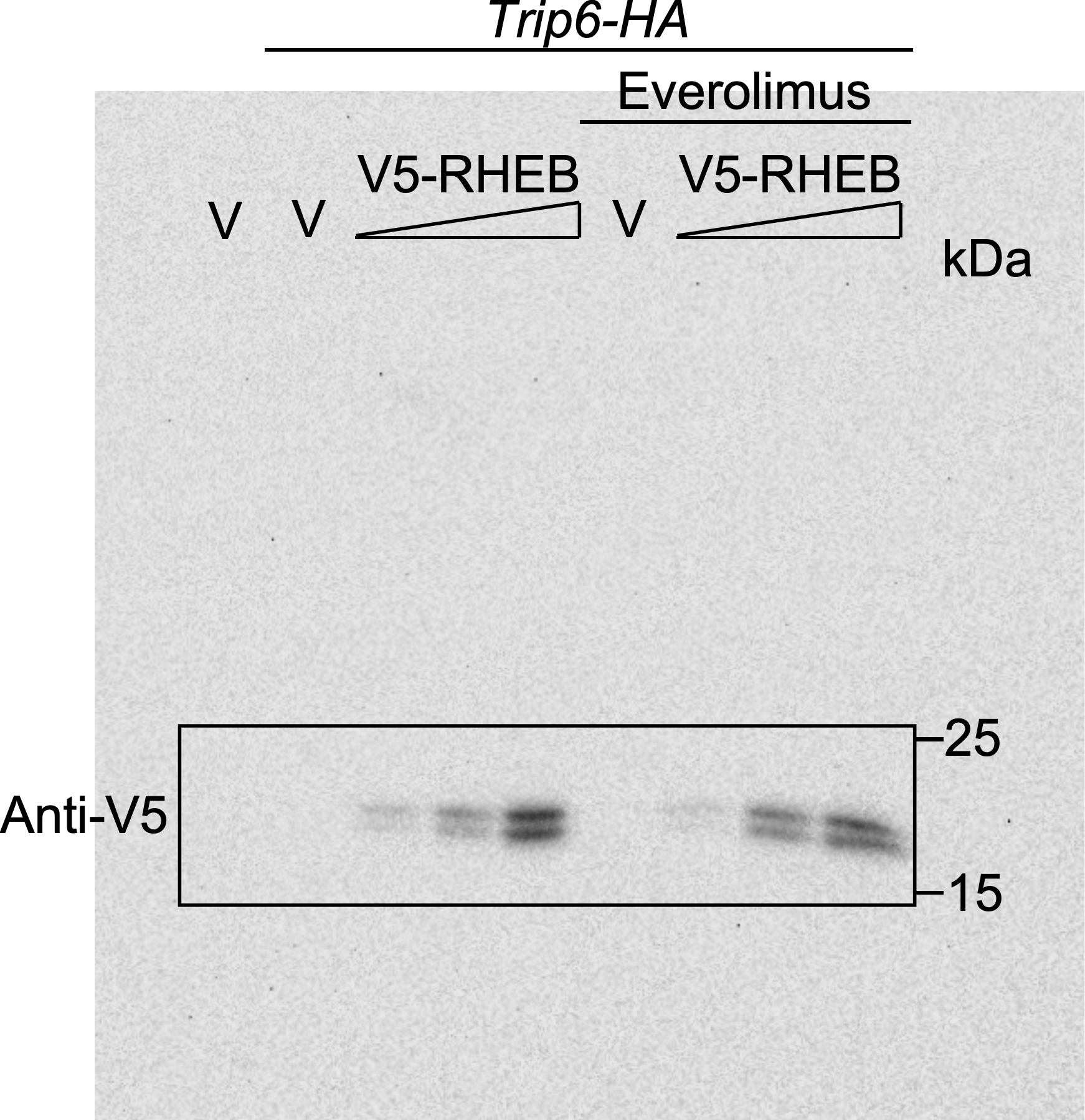

Supplement: Supplementary file 8 — Source data Fig. 4 [file 44319_2025_390_MOESM8_ESM.zip › Figure 4/4D/western_V5.tif]

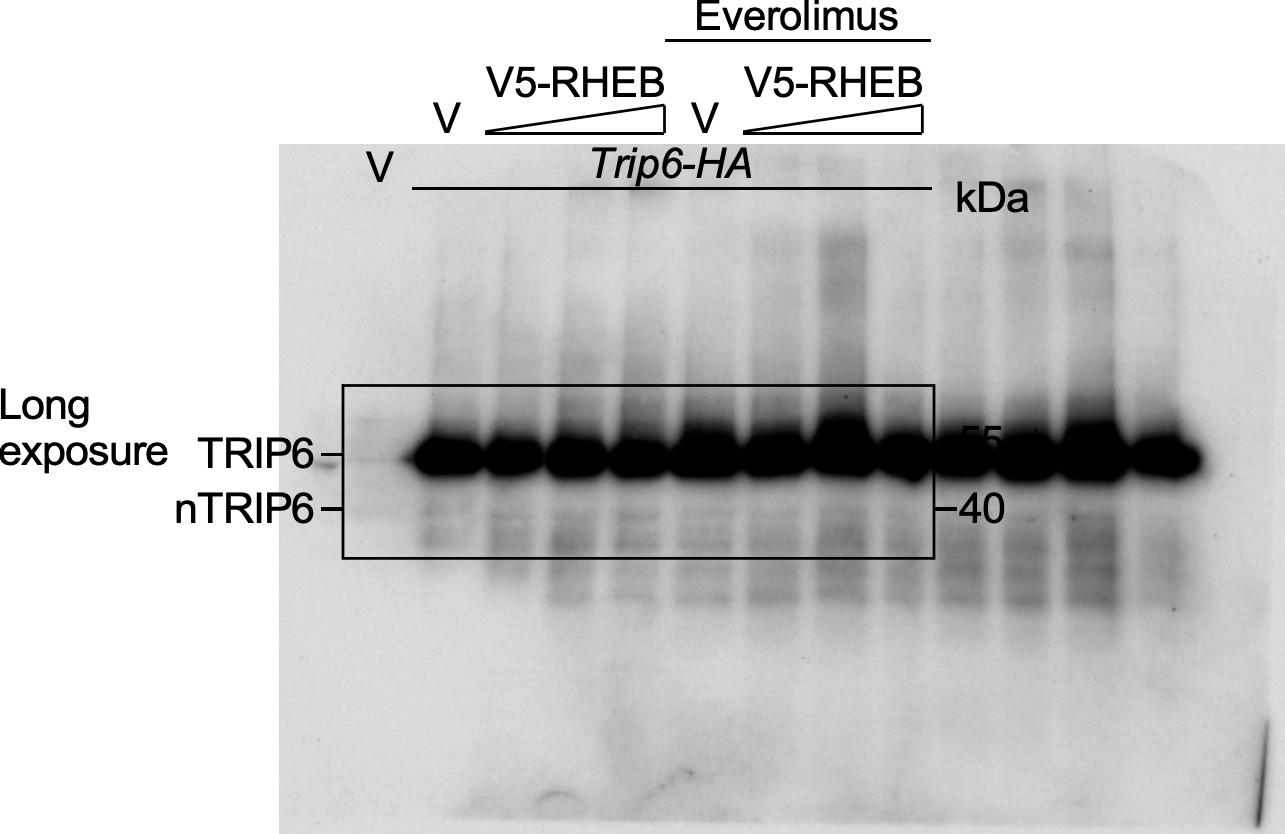

Supplement: Supplementary file 8 — Source data Fig. 4 [file 44319_2025_390_MOESM8_ESM.zip › Figure 4/4D/western_TRIP6_long_exposure.tif]

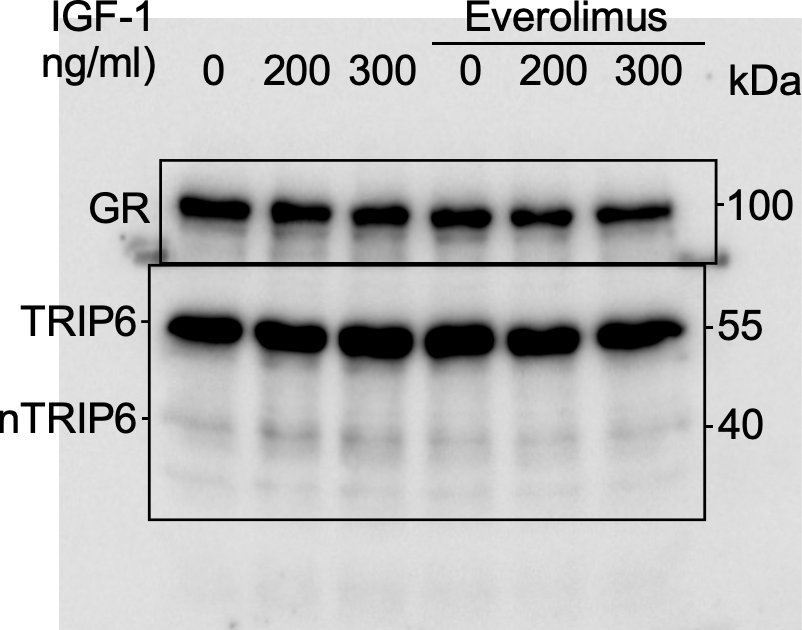

Supplement: Supplementary file 8 — Source data Fig. 4 [file 44319_2025_390_MOESM8_ESM.zip › Figure 4/4A/western_TRIP6_GR.tif]

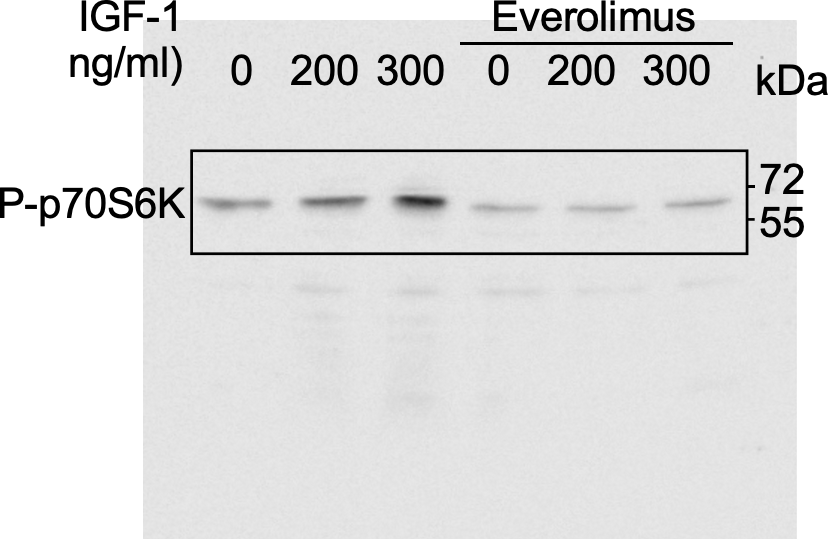

Supplement: Supplementary file 8 — Source data Fig. 4 [file 44319_2025_390_MOESM8_ESM.zip › Figure 4/4A/western_phosphoP70S6 kinase.tif]

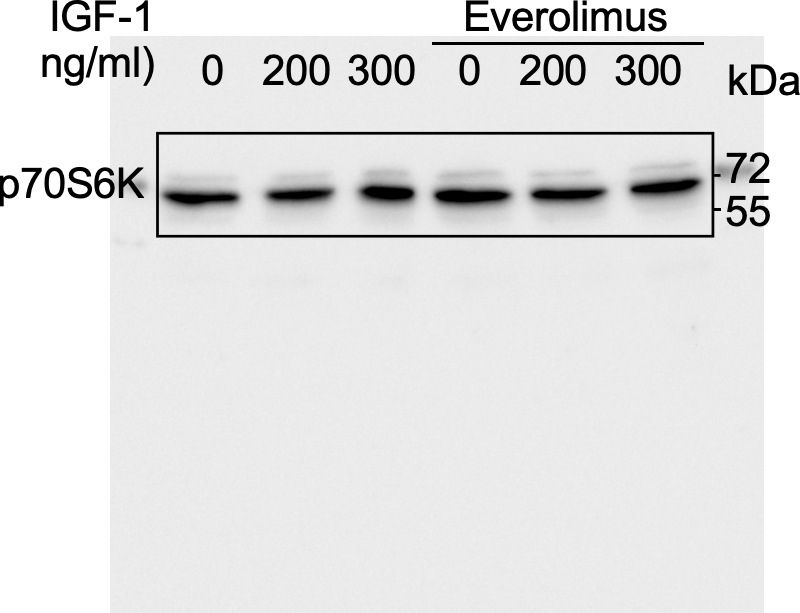

Supplement: Supplementary file 8 — Source data Fig. 4 [file 44319_2025_390_MOESM8_ESM.zip › Figure 4/4A/western_p70S6 kinase.tif]

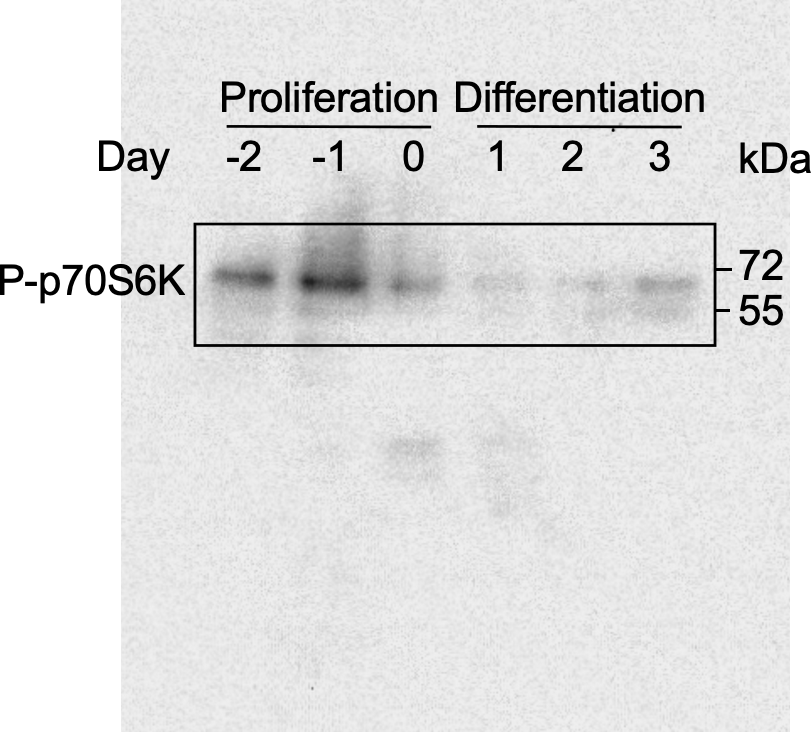

Supplement: Supplementary file 8 — Source data Fig. 4 [file 44319_2025_390_MOESM8_ESM.zip › Figure 4/4F/western_phosphoP70S6 kinase.tif]

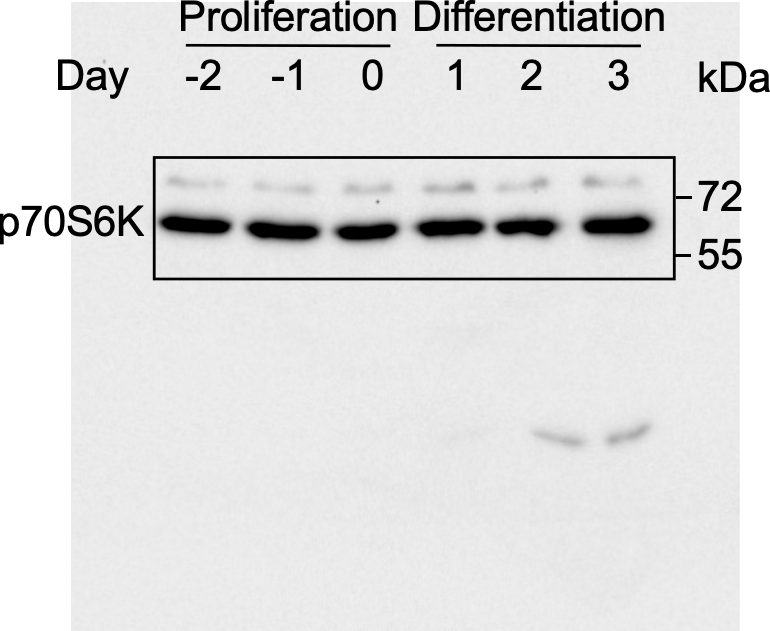

Supplement: Supplementary file 8 — Source data Fig. 4 [file 44319_2025_390_MOESM8_ESM.zip › Figure 4/4F/western_p70S6 kinase.tif]

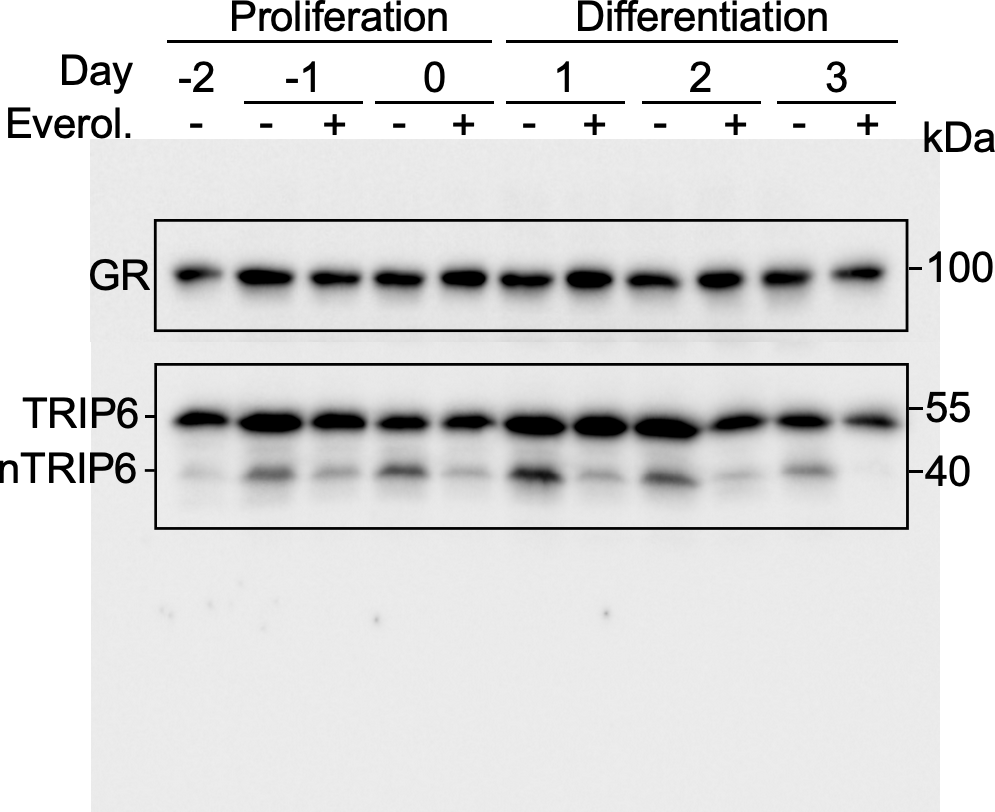

Supplement: Supplementary file 8 — Source data Fig. 4 [file 44319_2025_390_MOESM8_ESM.zip › Figure 4/4H/western_TRIP6_GR.tif]

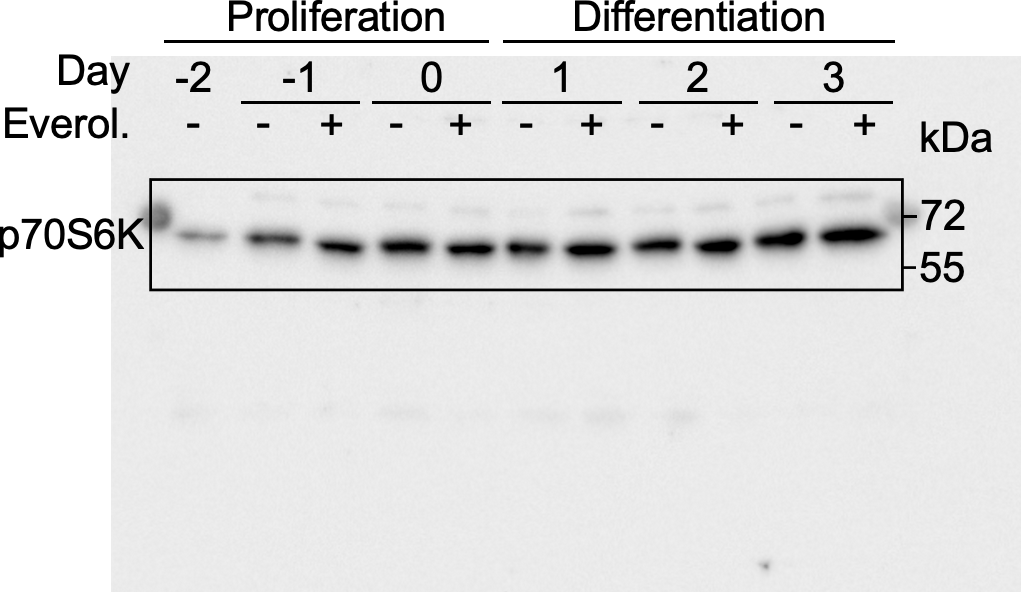

Supplement: Supplementary file 8 — Source data Fig. 4 [file 44319_2025_390_MOESM8_ESM.zip › Figure 4/4H/western p70S6 kinase.tif]

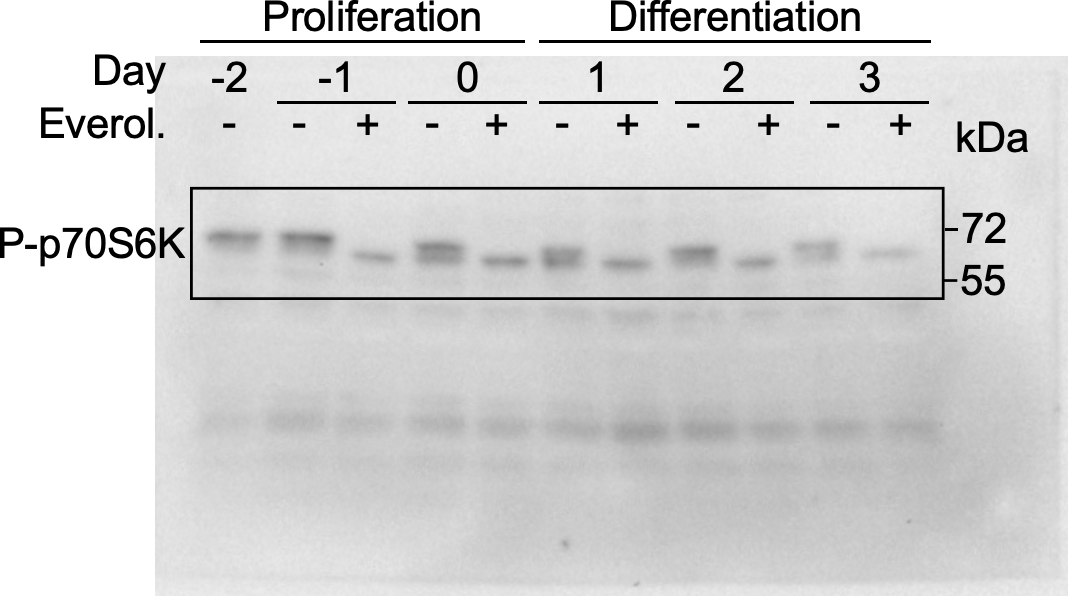

Supplement: Supplementary file 8 — Source data Fig. 4 [file 44319_2025_390_MOESM8_ESM.zip › Figure 4/4H/western phospho-p70S6 kinase.tif]

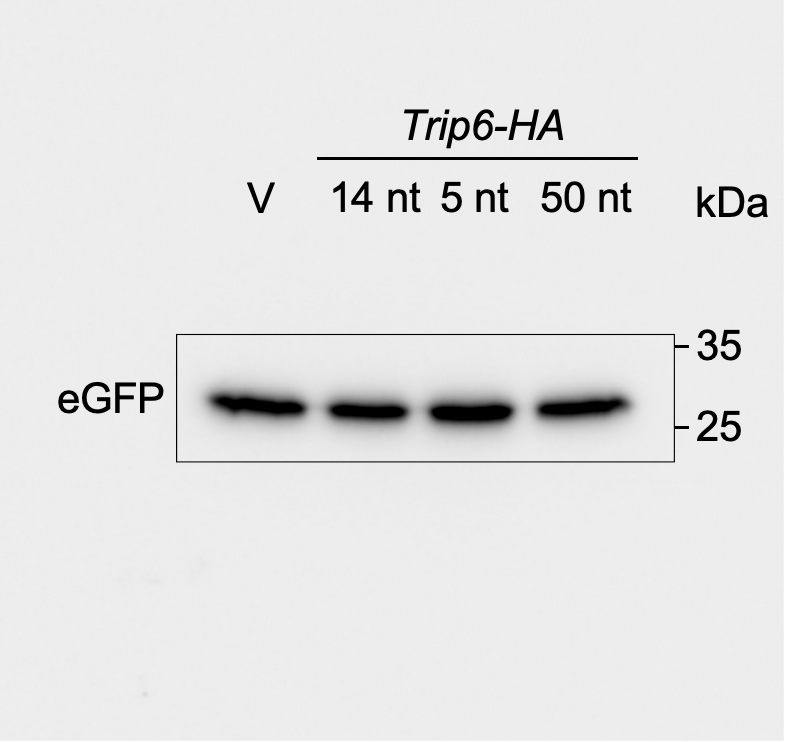

Supplement: Supplementary file 9 — Source data Fig. 5 [file 44319_2025_390_MOESM9_ESM.zip › Figure 5/5H/western eGFP.tif]

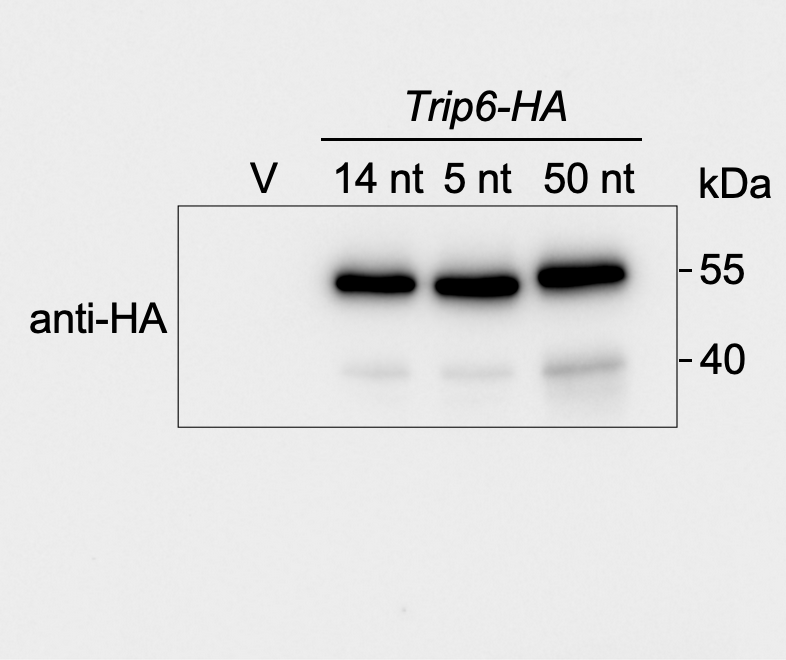

Supplement: Supplementary file 9 — Source data Fig. 5 [file 44319_2025_390_MOESM9_ESM.zip › Figure 5/5H/western HA.tif]

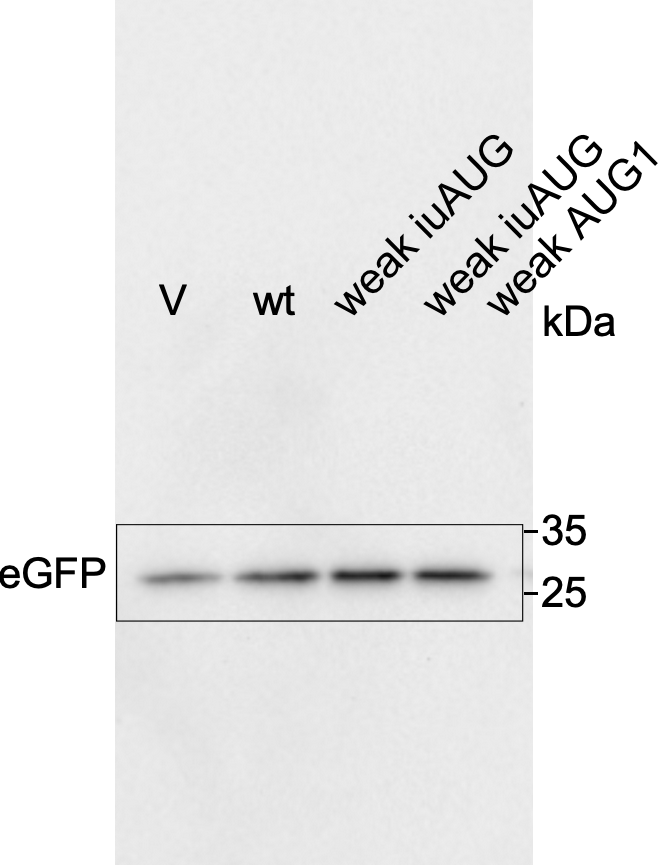

Supplement: Supplementary file 9 — Source data Fig. 5 [file 44319_2025_390_MOESM9_ESM.zip › Figure 5/5E/westen eGFP.tif]

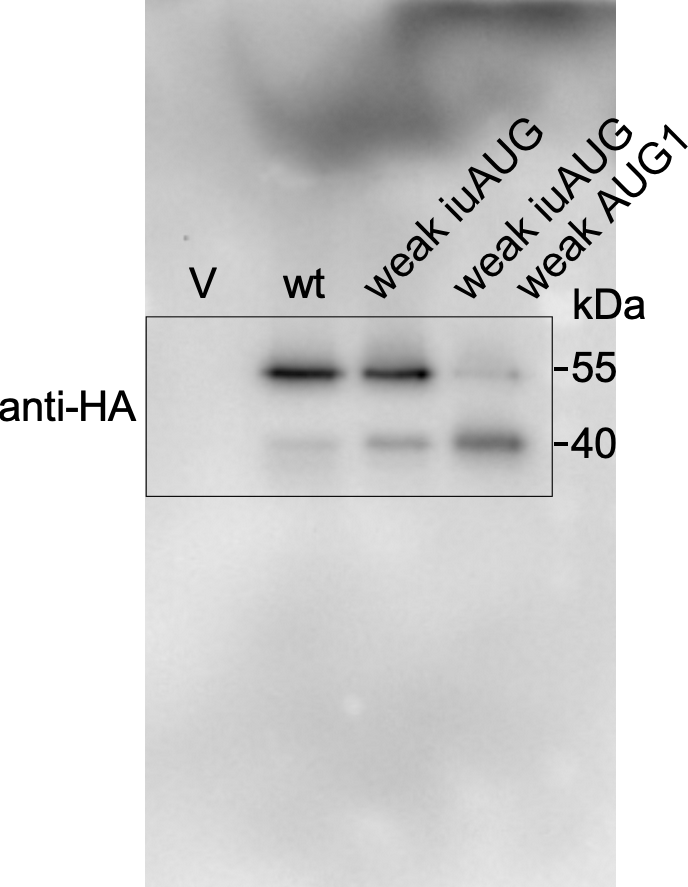

Supplement: Supplementary file 9 — Source data Fig. 5 [file 44319_2025_390_MOESM9_ESM.zip › Figure 5/5E/western HA.tif]

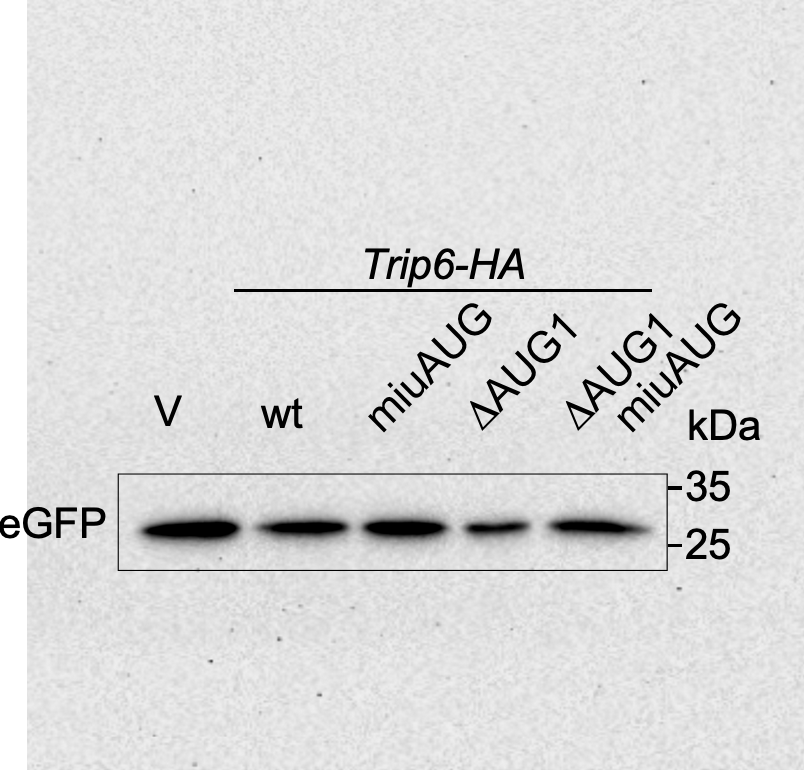

Supplement: Supplementary file 9 — Source data Fig. 5 [file 44319_2025_390_MOESM9_ESM.zip › Figure 5/5B/western eGFP.tif]

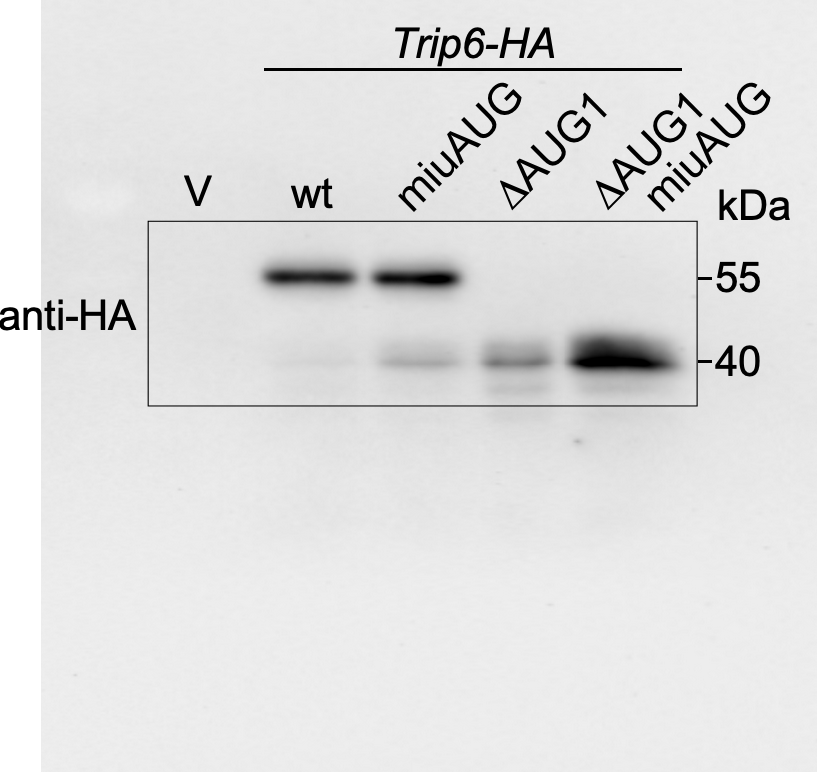

Supplement: Supplementary file 9 — Source data Fig. 5 [file 44319_2025_390_MOESM9_ESM.zip › Figure 5/5B/western HA.tif]

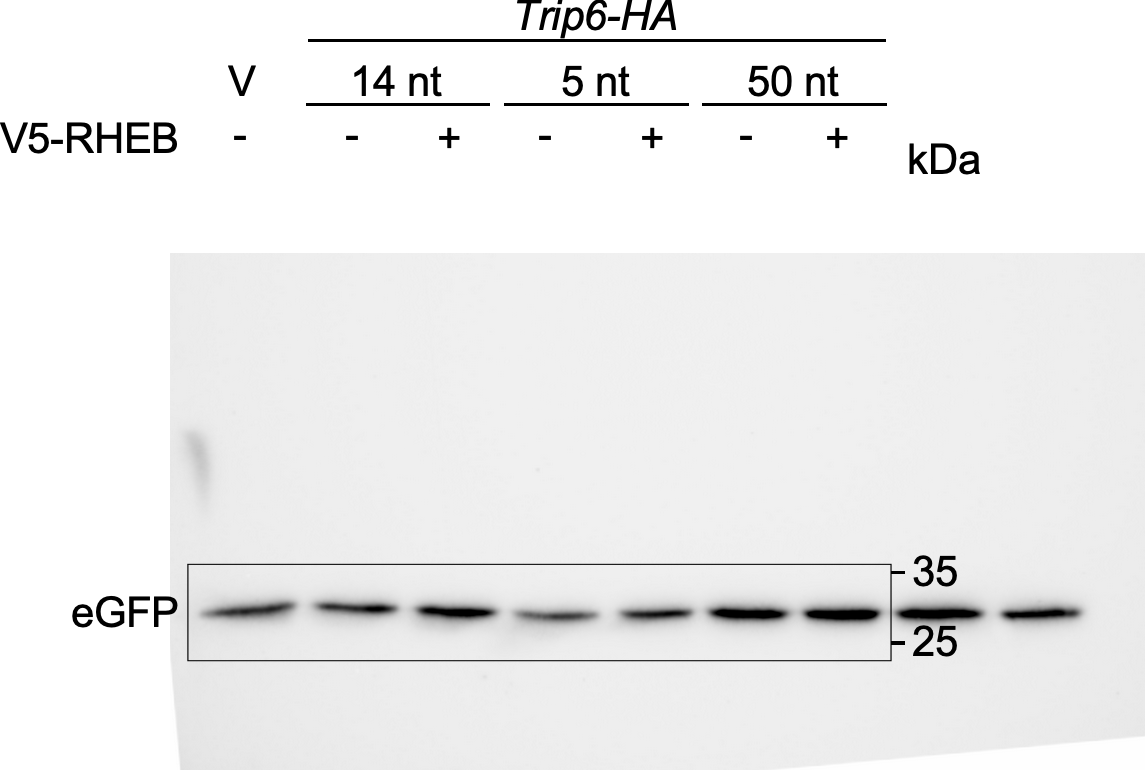

Supplement: Supplementary file 10 — Source data Fig. 6 [file 44319_2025_390_MOESM10_ESM.zip › Figure 6/6A/western eGFP.tif]

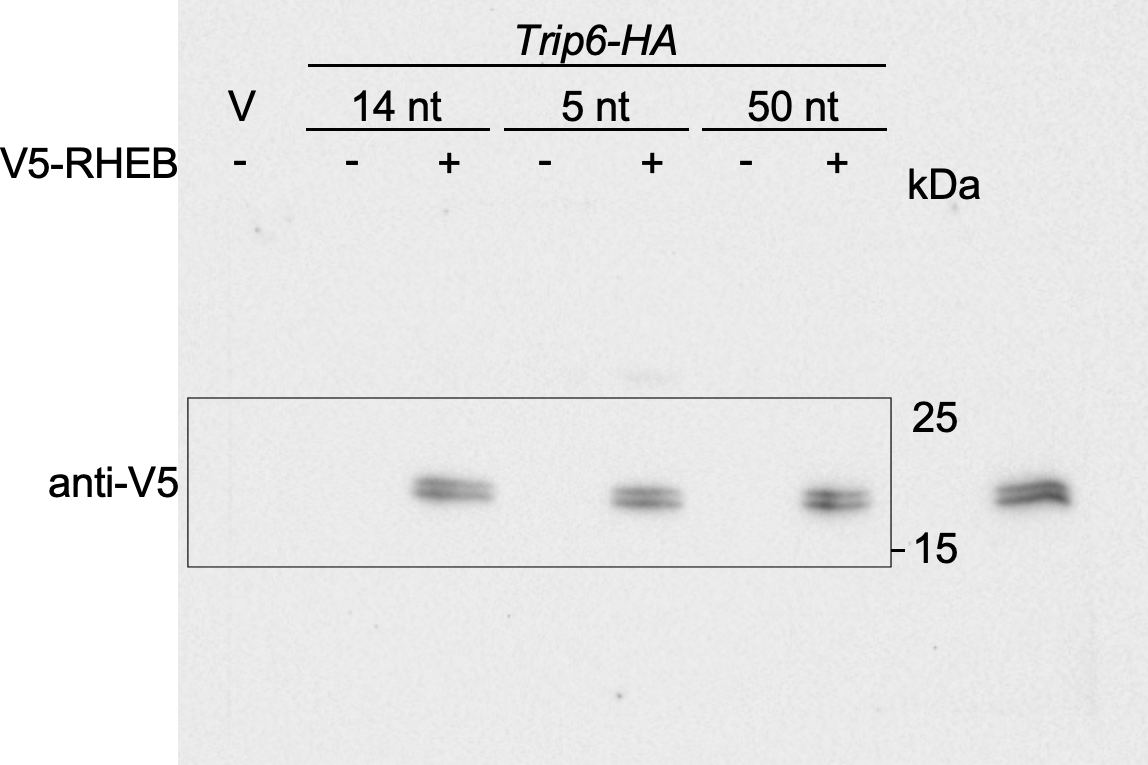

Supplement: Supplementary file 10 — Source data Fig. 6 [file 44319_2025_390_MOESM10_ESM.zip › Figure 6/6A/western V5.tif]

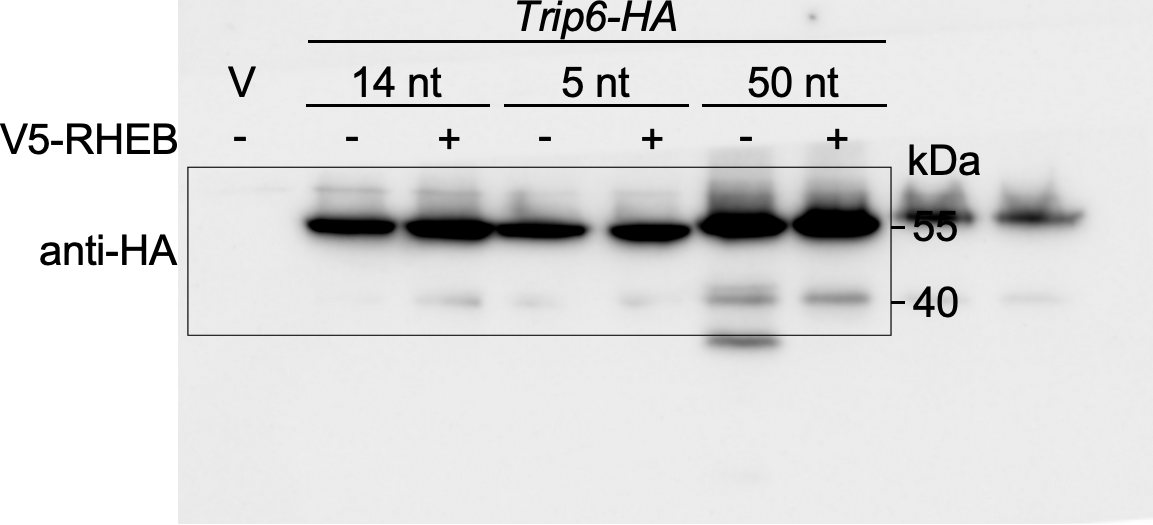

Supplement: Supplementary file 10 — Source data Fig. 6 [file 44319_2025_390_MOESM10_ESM.zip › Figure 6/6A/western HA.tif]

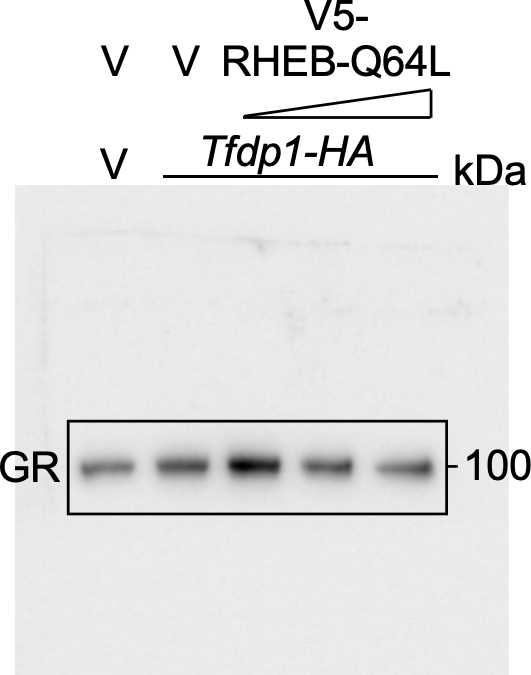

Supplement: Supplementary file 11 — Source data Fig. 8 [file 44319_2025_390_MOESM11_ESM.zip › Figure 8/8I/western GR.tif]

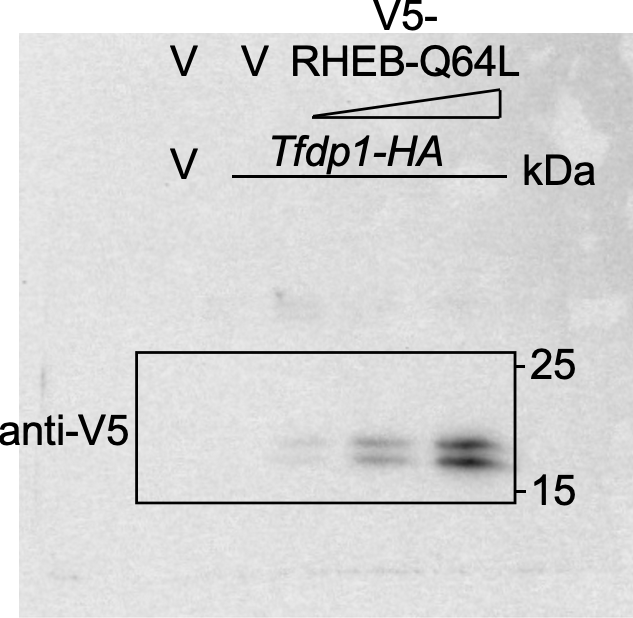

Supplement: Supplementary file 11 — Source data Fig. 8 [file 44319_2025_390_MOESM11_ESM.zip › Figure 8/8I/western V5.tif]

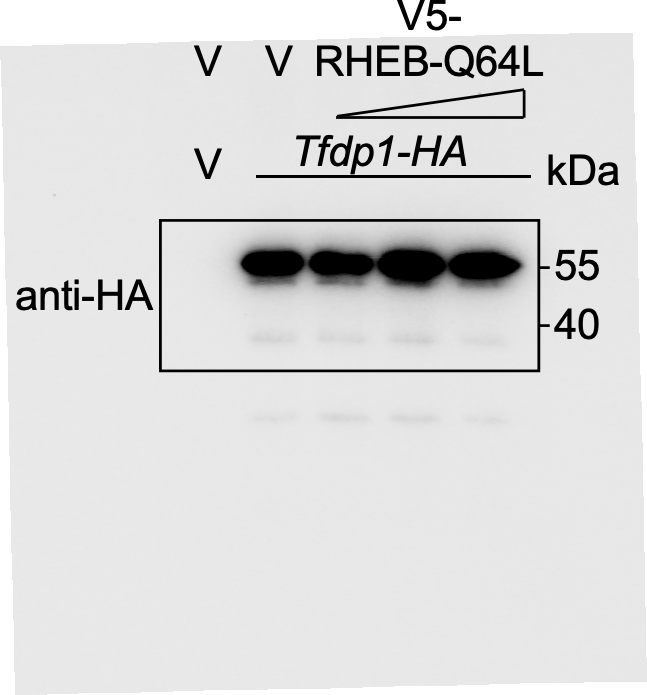

Supplement: Supplementary file 11 — Source data Fig. 8 [file 44319_2025_390_MOESM11_ESM.zip › Figure 8/8I/western HA.tif]

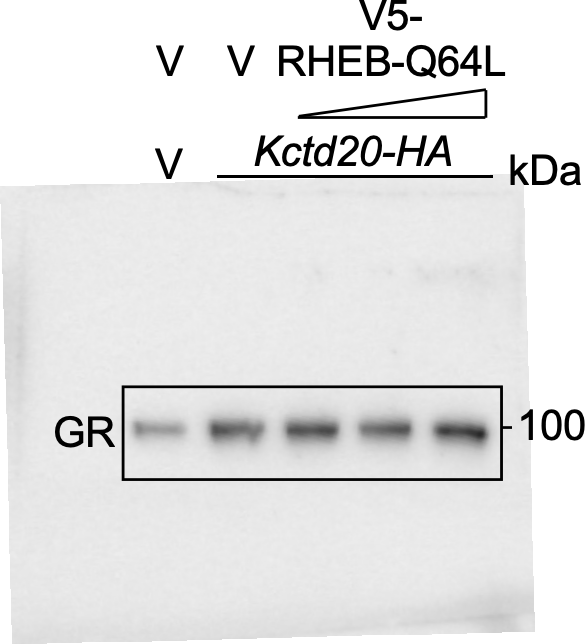

Supplement: Supplementary file 11 — Source data Fig. 8 [file 44319_2025_390_MOESM11_ESM.zip › Figure 8/8G/western GR.tif]

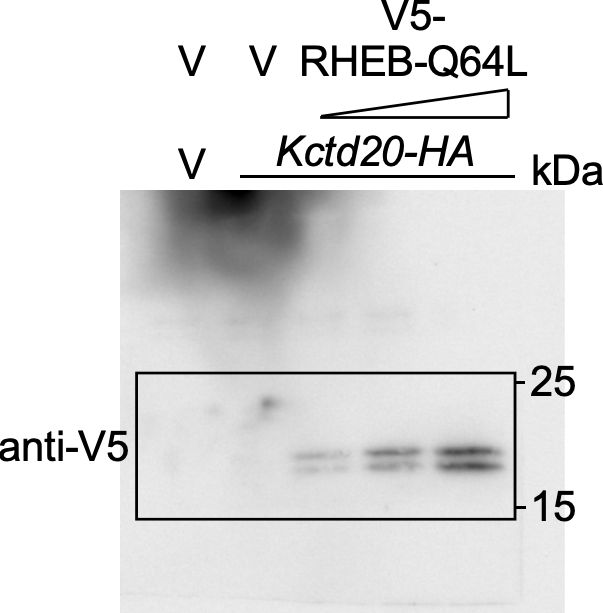

Supplement: Supplementary file 11 — Source data Fig. 8 [file 44319_2025_390_MOESM11_ESM.zip › Figure 8/8G/western V5.tif]

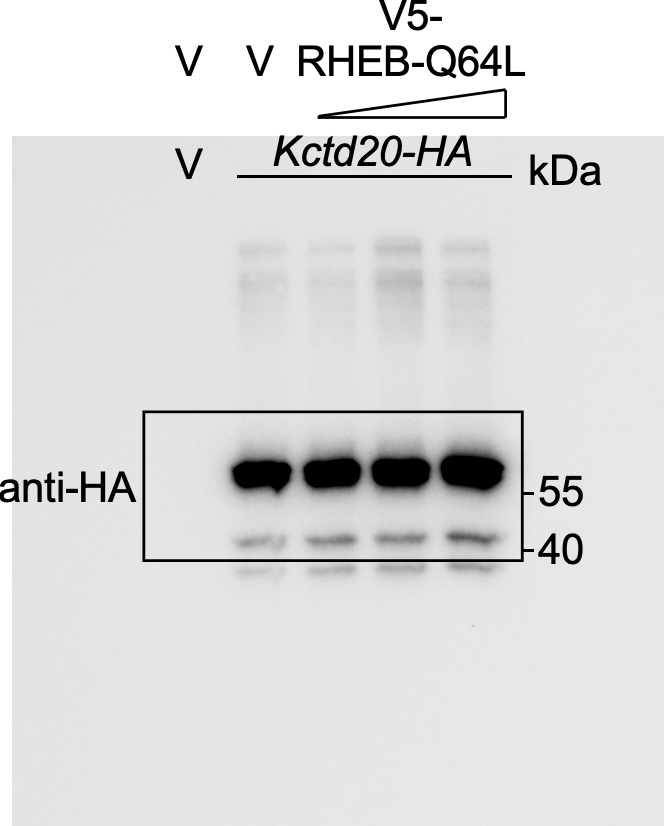

Supplement: Supplementary file 11 — Source data Fig. 8 [file 44319_2025_390_MOESM11_ESM.zip › Figure 8/8G/western HA.tif]

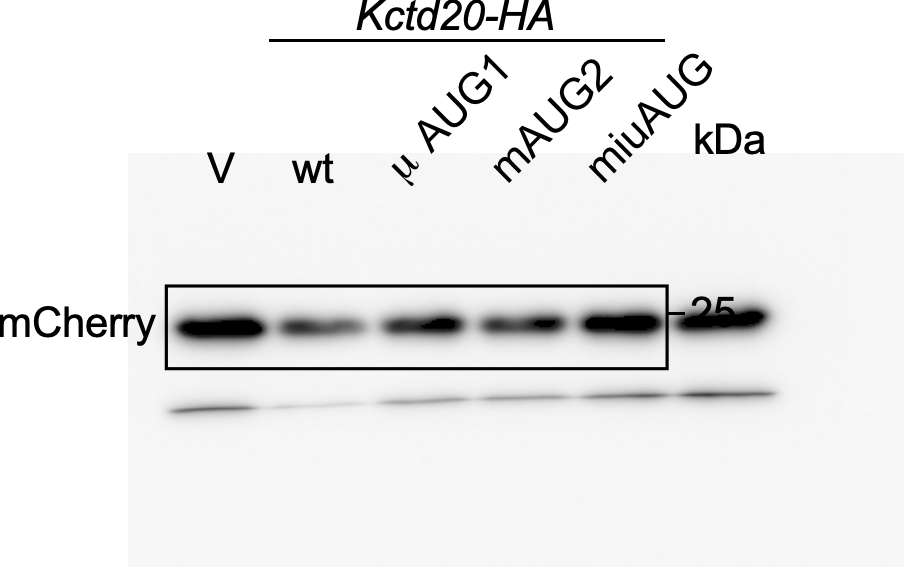

Supplement: Supplementary file 11 — Source data Fig. 8 [file 44319_2025_390_MOESM11_ESM.zip › Figure 8/8B/western mCherry.tif]

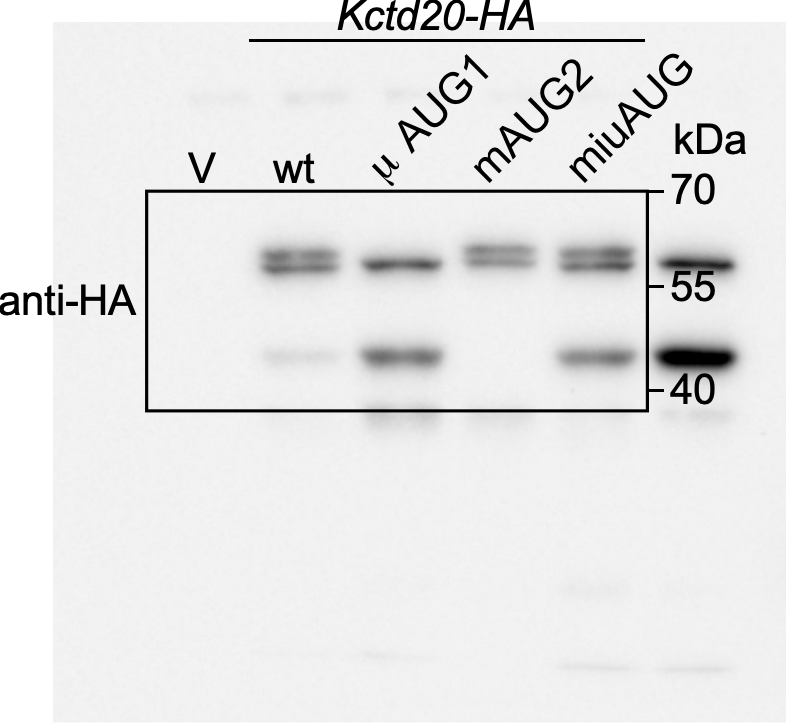

Supplement: Supplementary file 11 — Source data Fig. 8 [file 44319_2025_390_MOESM11_ESM.zip › Figure 8/8B/western HA.tif]

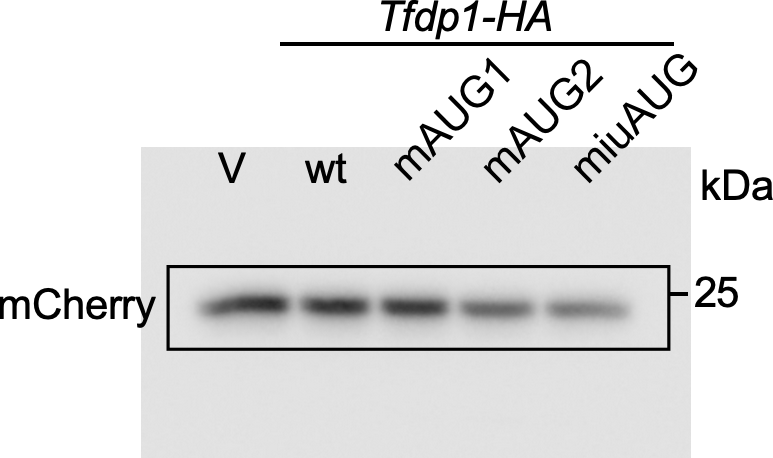

Supplement: Supplementary file 11 — Source data Fig. 8 [file 44319_2025_390_MOESM11_ESM.zip › Figure 8/8E/western mCherry.tif]

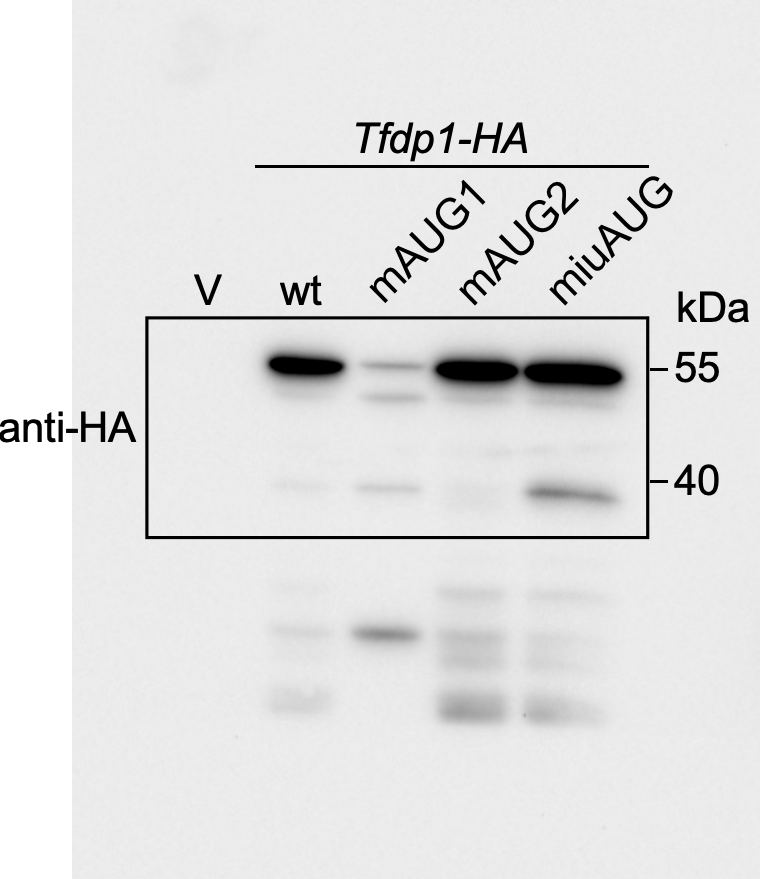

Supplement: Supplementary file 11 — Source data Fig. 8 [file 44319_2025_390_MOESM11_ESM.zip › Figure 8/8E/western HA.tif]
