# Supplementary material for: Short internal open reading frames repress the translation of N-terminally truncated proteoforms
Source: EMBO Rep. 2025 Feb 17;26(6):1566–89. doi: 10.1038/s44319-025-00390-z (PMC11933307; doi:10.1038/s44319-025-00390-z)
Supplement: Supplementary file 13 — Expanded View Figures [file 44319_2025_390_MOESM13_ESM.pdf]

Expanded View Figures

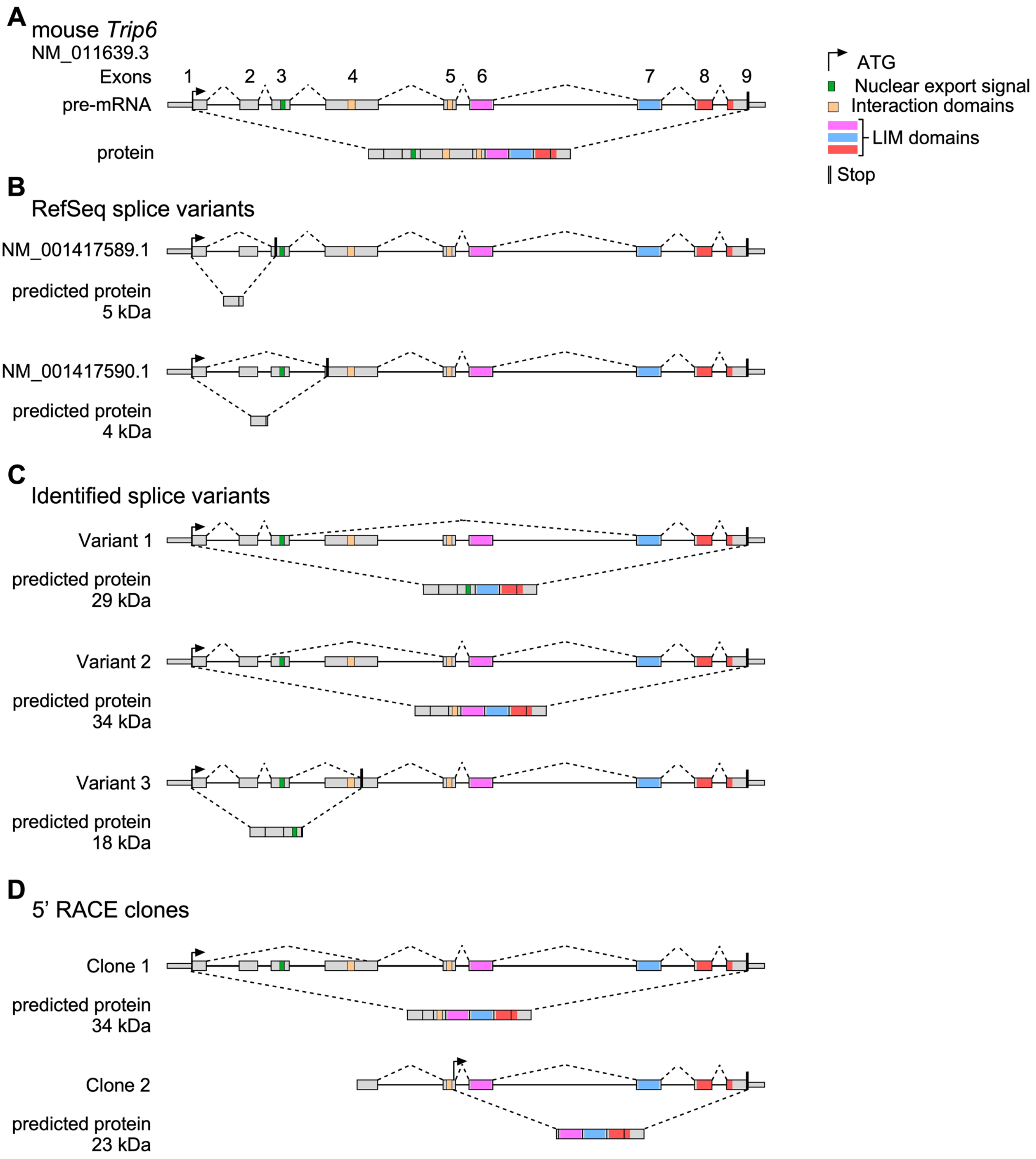

Figure EV1. Schematic representation of the identified *Trip6* mRNA variants.

(**A**) Exon/intron structure of mouse *Trip6* pre-mRNA and functional domain of TRIP6 protein. (**B–D**) Schematic representation of the published (**B**) or identified (**C, D**) *Trip6* pre-mRNA variants with the predicted translation products domain structure and molecular weight.

© The Author(s)

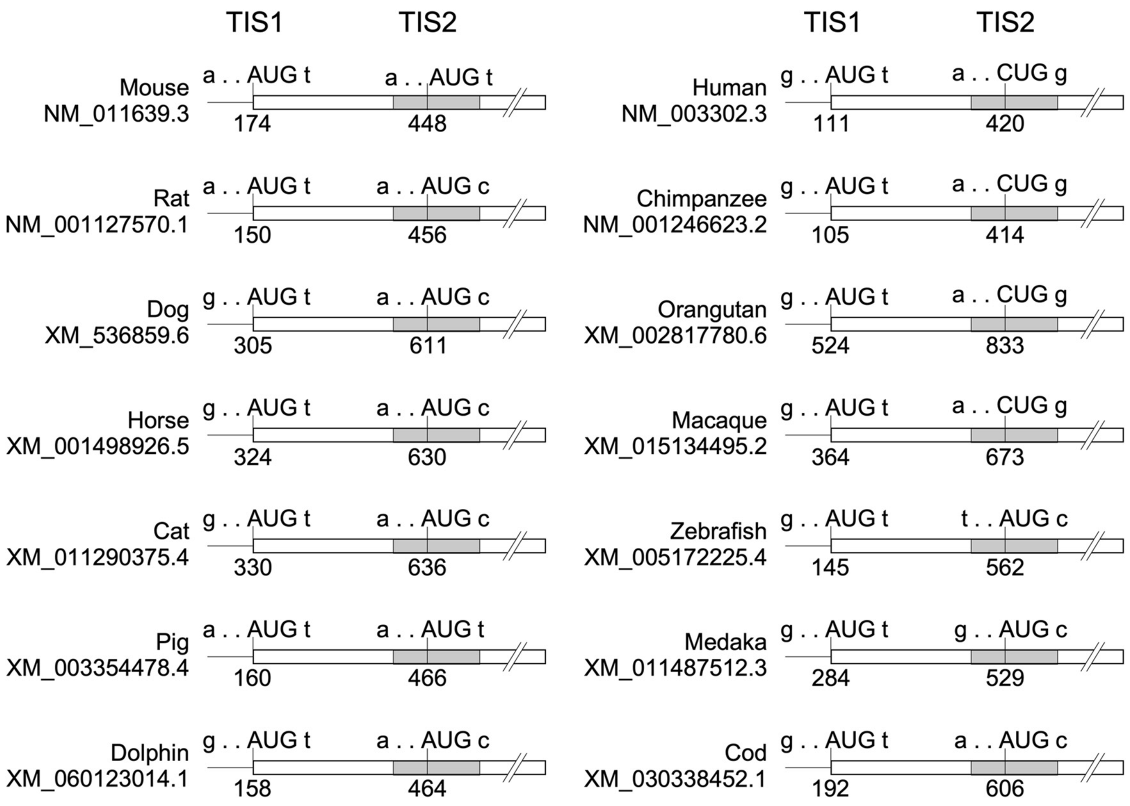

Figure EV2. Conservation of TRIP6 and nTRIP6 translation initiation sites in *Trip6* mRNA.

For each species, the schematic representation of the *Trip6* mRNA depicts the sequences surrounding the translation initiation site of TRIP6 (TIS1) and of nTRIP6 (TIS2) relative to the Kozak sequence (RNN AUG GNN where N represents any nucleotide and R a purine). The numbers indicate the positions on the mRNA. The gray box represents the Nuclear Export Signal encoding sequence.

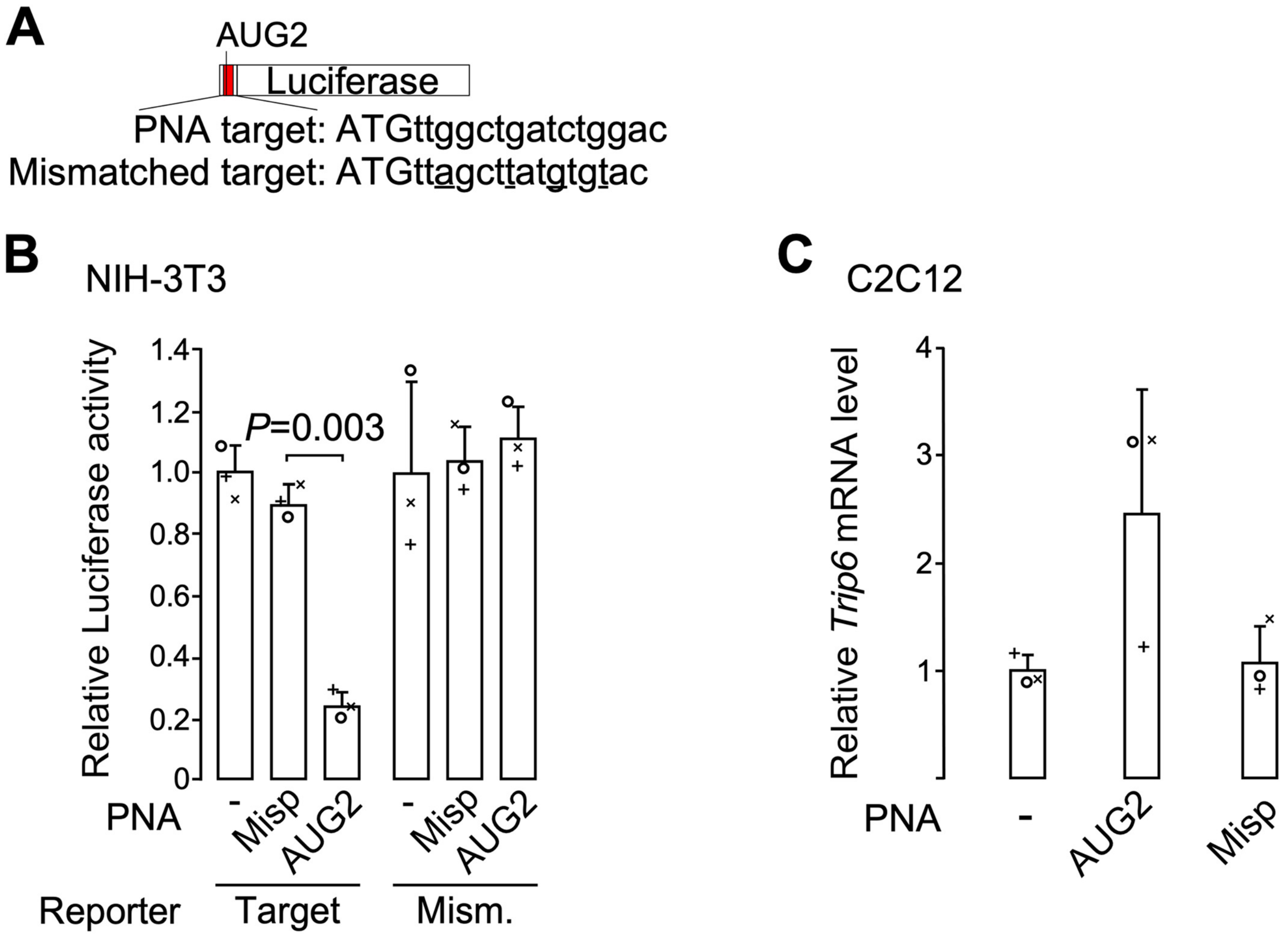

Figure EV3. Validation of the AUG2-targeting PNA.

(**A**) Schematic representation of the reporter constructs used. The PNA target sequence encompassing AUG2 (uppercase ATG) or a mismatched sequence (underlined mismatches) was fused in-frame with Firefly luciferase. (**B**) NIH-3T3 fibroblasts were transfected with the target or mismatched (Mism.) reporter construct together with an expression vector for Renilla luciferase and 4 h later mock-treated (-) or treated with the mispaired control PNA (Misp.) or the AUG2-targeting PNA. Normalized luciferase activities were determined 24 h later and are plotted relative to the target reporter transfected, mock-treated cells (mean ± SD of three independent experiments). (**C**) C2C12 myoblasts were mock-treated or treated with the indicated PNA for 24 h. Relative levels of *Trip6* mRNA were determined by reverse transcription and real-time PCR. Results are plotted relative to the expression of the *Rplp0* gene (mean ± SD of three independent experiments). In each graph individual values are depicted by symbols, each representing an independent experiment. Student's paired *t* test *P* values are indicated. Source data are available online for this figure.

© The Author(s)

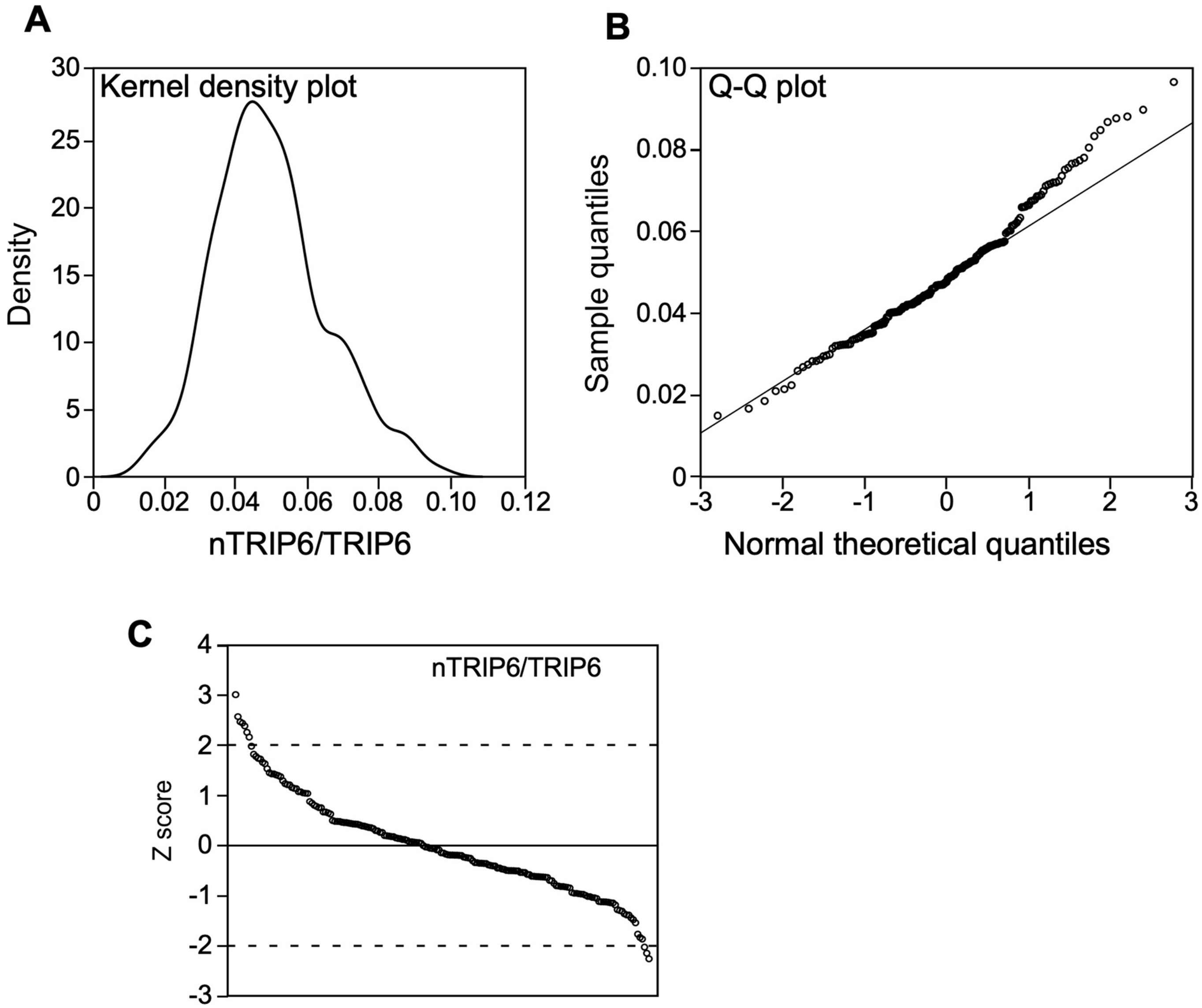

Figure EV4. Relative expression of nTRIP6 and TRIP6 in the kinase overexpression screen.

The nTRIP6/TRIP6 ratios were determined by western blotting analysis in HEK-293 co-transfected with the C-terminally V5-tagged Trip6 CDS construct and a library of 184 unique Medaka kinases. (**A**, **B**) The ratios are normally distributed. The Kernel density and Q-Q plots are presented. Shapiro–Wilk normality test: $W = 0.9803$, $P = 1.07 \times 10\text{-}2$. (**C**) Z scores of the nTRIP6/TRIP6 ratios.

© The Author(s)

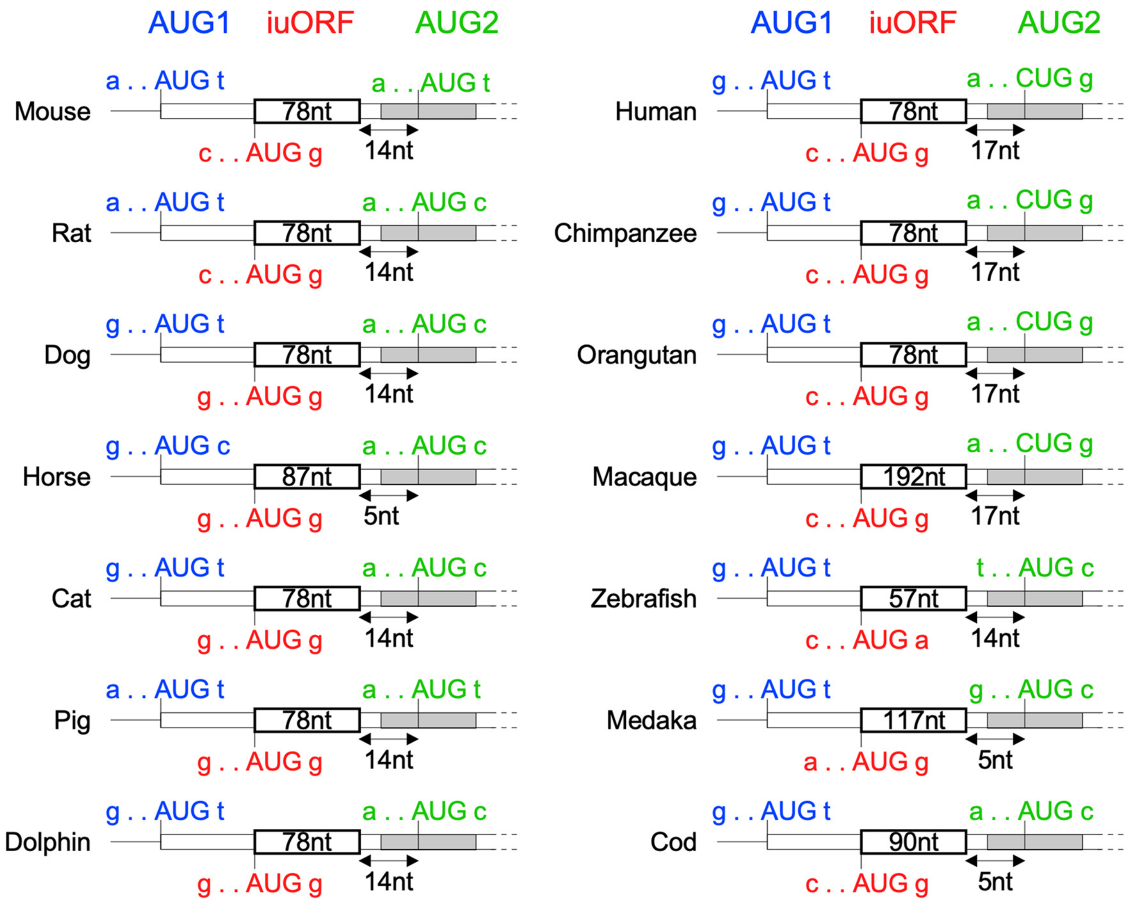

Figure EV5. Conservation of the internal upstream open reading frame (iuORF) in Trip6 mRNA.

For each species, the schematic representation of the 5′ of Trip6 mRNA depicts the surrounding sequence of the TRIP6 (AUG1), iuORF and nTRIP6 (AUG2) initiation codons relative to the Kozak sequence (RNN AUG GNN where N represents any nucleotide and R a purine), the number of nucleotides (nt) in the uORF and the distance between the iuORF and AUG2. The gray box represents the Nuclear Export Signal encoding sequence.

 © The Author(s)
